# Genome-Wide Identification of MAPKK and MAPKKK Gene Families in Tomato and Transcriptional Profiling Analysis during Development and Stress Response

**DOI:** 10.1371/journal.pone.0103032

**Published:** 2014-07-18

**Authors:** Jian Wu, Jie Wang, Changtian Pan, Xiaoyan Guan, Yan Wang, Songyu Liu, Yanjun He, Jingli Chen, Lifei Chen, Gang Lu

**Affiliations:** Key Laboratory of Horticultural Plant Growth, Development and Biotechnology, Agricultural Ministry of China, Department of Horticulture, Zhejiang University, Hangzhou, People’s Republic of China; Pennsylvania State University, United States of America

## Abstract

Mitogen-activated protein kinase (MAPK) cascades have important functions in plant growth, development, and response to various stresses. The MAPKK and MAPKKK gene families in tomato have never been systematically analyzed. In this study, we performed a genome-wide analysis of the MAPKK and MAPKKK gene families in tomato and identified 5 MAPKK genes and 89 MAPKKK genes. Phylogenetic analyses of the MAPKK and MAPKKK gene families showed that all the MAPKK genes formed four groups (groups A, B, C, and D), whereas all the MAPKKK genes were classified into three subfamilies, namely, MEKK, RAF, and ZIK. Evolutionary analysis showed that whole genome or chromosomal segment duplications were the main factors responsible for the expansion of the MAPKK and MAPKKK gene families in tomato. Quantitative real-time RT-PCR analysis showed that the majority of MAPKK and MAPKKK genes were expressed in all tested organs with considerable differences in transcript levels indicating that they might be constitutively expressed. However, the expression level of most of these genes changed significantly under heat, cold, drought, salt, and *Pseudomonas syringae* treatment. Furthermore, their expression levels exhibited significant changes in response to salicylic acid and indole-3-acetic acid treatment, implying that these genes might have important roles in the plant hormone network. Our comparative analysis of the MAPKK and MAPKKK families would improve our understanding of the evolution and functional characterization of MAPK cascades in tomato.

## Introduction

Mitogen-activated protein kinase (MAPK) cascades, which are widely distributed in eukaryotes, have an important function in the diverse developmental and physiological processes of plants, and in response to various biotic and abiotic stresses [1], [2]. Each MAPK cascade consists of three protein kinases: MAPKs, MAPK kinases (MAPKKs/MKKs), and MAPKK kinases (MAPKKKs/MEKKs). MAPKKK activates MAPKK through the phosphorylation of serine and serine/threonine residues in its T-loop. Similarly, MAPKK activates MAPK through the phosphorylation of tyrosine and threonine residues in the TEY or TDY motif between kinase subdomains VII and VIII. Plant MAPK cascade genes were first reported in *Arabidopsis*. Up to now, MAPK cascade family genes have been identified in many other plant species, including poplar, rice, soybean, maize, tomato and *N. benthamiana* [3]–[12].

In plants, the number of MAPKK family genes varies markedly across species. The estimated number is 10 in *Arabidopsis*, eight in rice, 11 in soybean, 11 in poplar and 12 in *Brachypodium distachyon*. According to phylogenetic analyses, all MAPKKs can be classified into four groups (groups A–D). The function of the group A MAPKK members, including *AtMAPKK1*, *AtMAPKK2*, and *AtMAPKK6*, in *Arabidopsis* has been detailed studied. *AtMAPKK2* is shown to play an important role not only mediates innate immunity responses but also has an important function in the cold and salt signaling transduction pathway [13], [14]. *AtMAPKK1* also has essential functions in pathogen defence and have functional redundancy with *AtMAPKK2* [13], [15]. *AtMAPKK6* acts in upstream of *AtMAPK13* in yeast cells [16]. Functional data on MAPKK family members belonging to the other three subgroups are limited. Only one *AtMAPKK3* belonging to group B was proved to function in pathogen resistance and participate in jasmonate signal transduction pathway in *Arabidopsis* [17], [18].

MAPKKK is a large gene family containing 80, 75, 74 and 150 members in *Arabidopsis*, rice, maize and soybean, respectively [7], [19]–[21]. All MAPKKK genes have been divided into three major groups, namely, RAF, MEKK, and ZIK. The RAF subfamily in rice, maize, and *Arabidopsis* has 48, 43, and 46 members, respectively [20]–[22]. This subfamily contains a conserved catalytic and RAF-specific signature GTXX (W/Y) MAPE [22]. Plant MEKK-like MAPKKK genes also harbor a conserved catalytic domain and conserved signature G (T/S) PX (F/Y/W) MAPEV, similar to animal MEKKs. The ZIK subfamily contains a conserved signature GTPEFMAPE (L/V/M) (Y/F/L) across these members. Functional data on MEKK-like genes are more readily available than that on the other two subfamilies. In *Arabidopsis*, three MAPKKK genes named *ANP1*, *ANP2*, and *ANP3* have an important function in the signal transduction pathways that control plant cell division [23], [24]. Another *Arabidopsis* MEKK-like gene (MEKK1) is involved in stress response and participates in signal transduction in diverse development process [25], [26]. A MAPKKK gene, *YODA* regulates stomatal development in *Arabidopsis* [27]. The functional characteristics of MEKK-like genes from other species, such as tobacco and *Solanum chacoense* have been studied in depth [28], [29]. In tomato, *SlMAPKKKα* positively regulates cell death associated with both plant immunity and disease resistance [30]. *SlMAPKKKε* is involved in signaling networks associated with plant immunity [31]. The functions of some RAF subfamily members have also been investigated in *Arabidopsis* and other plant species [3]. *CTR1* and *EDR1*, belonging to the *Arabidopsis* RAF MAPKKK subfamily, negatively regulates ethylene signaling transduction and participates in pathogen resistance [32], [33]. *MAP3Kδ4,* an *Arabidopsis* Raf-like MAP3K, has a function in the regulation of plant growth and shoot branching [34]. A rice RAF-like MAPKKK named ILA1 regulates mechanical tissue formation [35]. *DSM1* is involved in rice drought resistance [36]. Function analysis of ZIK-like genes is limited. However, most rice ZIK-like MAPKKK genes can be upregulated by at least one abiotic stress [20], indicating that they might be involved in stress signaling transduction pathways.

Up to now, only four MAPKK genes (*SlMAPKK1-4*) [37] and three MAPKKK genes (*MAPKKKα*, *MAPKKKε*, and *NPK1*) [31], [38], [39] in tomato have been identified. The three gene families that involved in MAPK cascade have never been systematically investigated in tomato except in our recent report on the MAPK gene family [11]. Taking advantage of the available tomato genome database, we performed a genome-wide search for the homologues of the MAPKK and MAPKKK families in tomato. Detailed information on the genomic structures, chromosomal locations, and sequence homologies of these genes is presented in this paper. In addition, the phylogenetic relationships of these gene families in *Arabidopsis*, tomato, rice, and maize were compared. Finally, the expression profiles of SlMAPKK and SlMAPKKK genes during development and in response to various biotic and abiotic stress treatments were investigated through quantitative real-time reverse transcription PCR (qRT-PCR) analyses.

## Materials and Methods

### Searching for MAPKK and MAPKKK family genes

Predicted tomato peptide sequences were downloaded from the SGN database (http://solgenomics.net/organism/Solanum_lycopersicum/genome) to construct a local protein database. To identify tomato MAPKK, this database was searched using all known plant MAPKK protein sequences, including 10 AtMAPKKs, 8 OsMAPKKs, 11 GmMAPKKs and 11 PtMAPKKs, as query sequences downloaded from NCBI (http://ncbi.nlm.nih.gov), TAIR (http://www.arabidopsis.org), and a rice genome database (http://ftp.ncbi.nih.gov/genbank/genomes/Eukaryotes/plants/Oryza sativa/). Similarly, for the tomato MAPKKK gene family, 80 AtMAPKKK, 75 OsMAPKKK, and 74 ZmMAPKKK protein sequences from *Arabidopsis*, rice, and maize were used as query sequences to search against the tomato genome database [7], [20], [21]. The search was carried out using BLASTP, and 50% identity was used as the threshold for the sequences obtained from BLAST analysis. Self BLAST of the sequences was carried out to remove redundancies. The putative functional domains of all the sequences were detected by BLASTP of NCBI (http://blast.ncbi.nlm.nih.gov), and identified using the Pfam program under a default E-value level (0.01) (http://www.Pfam.sanger.ac.uk/) and the SMART database (http://smart.embl-heidelberg.de/). Sequences without known conserved domains of the MAPKK or MAPKKK gene families were excluded from further analysis. Finally, predictions of MAPKK and MAPKKK sequences were further verified with gene structure, EST, and unigene analyses. Furthermore, the full-length cDNA sequences of predicted MAPKKs and MAPKKKs in tomato were identified by BLASTN against the Kazusa Full-length Tomato cDNA Database (http://www.pgb.kazusa.or.jp/kaftom/blast.html). The isoelectric point (pI) of the MAPKK and MAPKKK proteins was predicted using Compute pI/Mw software (http://www.expa sych/tools/pitool.html). Subcellular localization prediction of each of these family genes was carried out using the CELLO v2.5 server (http://cello.life.nctu.edu.tw/) [40].

### Multiple sequence alignment and phylogenetic analysis

Multiple sequence alignment for all the MAPKKs and MAPKKKs in *Arabidopsis*, rice, maize, and tomato was generated using ClustalX v1.81 [41]. PlantsP (http://plantsp.genomics.purdue.edu/index.html) was used to scan the motifs and domains of these kinase protein sequences. Phylogenetic analysis was performed using MEGA 4.1 program by the neighbor-joining (NJ) method [42], and a bootstrap test was carried out with 1000 interactions based on the full-length protein sequences.

### Cis-element analysis of putative promoter regions of MAPKK and MAPKKK genes

To investigate cis-elements in the promoter regions of MAPKK and MAPKKK genes, 2000 bp of the genomic DNA sequences upstream of the transcriptional start site of each MAPKK and MAPKKK gene were chosen. These sequences were used to search against the PLACE database (http://www.dna.affrc.go.jp/PLACE/) to find the putative cis-regulatory elements.

### Mapping MAPKK and MAPKKK genes on chromosomes and gene duplications

To determine the location of tomato genes on chromosomes, the nucleotide sequences of all these genes were further used as query sequences for BLASTN search against SGN Tomato Whole Genome Scaffold data (2.30) (http://www.sgn.cornell.edu/tools/blast/). Finally, the locations of these genes in tomato were detected. Synteny analysis of the SlMAPKK and SlMAPKKK genes was performed online using PGDD (http://chibba.agtec.uga.edu/duplication/) [43]. Tandem duplications were defined as genes located within five loci of each other [44].

### Plant materials, growth conditions, and treatments

Tomato (*S. lycopersicum* L.) cv. Micro-Tom plants used for expression analysis from the Tomato Genetics Resource Center (University of California, Davis, USA) were grown in growth chambers at 26±1°C at 40% to 50% relative humidity with a photoperiod of 14 h light/10 h dark. Three-week-old seedlings with three fully opened leaves were used for all abiotic and biotic treatments. The leaves, stems, roots, flower buds (1 d before flowering), and fruits (10 d after pollination) were collected from flowering plants. All the samples were frozen in liquid nitrogen immediately and stored at –75°C until RNA isolation.

Heat and cold stresses were produced by incubating the seedlings at 37±1°C and 4±1°C for 2 h, respectively. Drought stress was initiated by withholding water supply to three-week-old seedlings after they were fully watered. Leaves were harvested after withholding water for 7 d when the leaves started to curl because of drought stress. Salt stress was produced by adding 200 mM sodium chloride to the planter box for 3 h. Control seedlings were grown at 26±1°C with normal irrigation.

Biotic stress treatment was carried out using *Pseudomonas syringae* pv. tomato DC3000 cultivated in King’s B medium. The cells were pelleted, resuspended, and diluted in 10 mM MgSO_4_ and 0.02% Silwet-77 to a concentration of 2×10^5^ CFU ml^–1^ to 8×10^6^ CFU ml^–1^. The plants were spray-inoculated until leaf surfaces were uniformly wet. Meanwhile, the control seedlings were sprayed with ddH_2_O with 10 mM MgSO_4_ and 0.02% Silwet-77 without bacterial strains. After inoculation, the tomato plants were incubated at 26±1°C in 60 % relative humidity with a 14 h photoperiod for the duration of the experiment. The samples were collected 2 h after treatment.

For hormone treatments, the seedling leaves were sprayed with 100 mM indole-3-acetic acid (IAA) or 100 mM salicylic acid (SA), and sampled at 0, 1, 2, 4, 8, and 16 h intervals [45].

Each experiment was repeated three times, and 20 seedlings were used in each replication of each treatment.

### RNA extraction and qRT-PCR expression analysis

The total RNA was extracted using TRIZOL reagent (Invitrogen, Germany) according to the manufacturer’s instructions. The first cDNA strand was generated using a Takara Reverse Transcription System (Japan) following the manufacturer’s protocol. A maximum of 1 µg of RNA was used for each reverse-transcription reaction, and a gDNA eraser in the kits was used to eliminate DNA to prevent DNA contamination. qRT-PCR techniques were employed to characterize the gene expression profiles of SlMAPKKs and SlMAPKKKs using the primer pairs designed by Applied Biosystems Primer Express software (Table S1). To ensure the specificity of each primer to its corresponding gene, the primers were submitted to the tomato genome database for BLAST search. All non-specific primers that show more 50% percent sequence similarity to multiple regions were eliminated and redesigned to minimize potential non-specific PCR amplification. Thus, the results from real-time PCR analysis might represent the expression pattern of a specific MAPKK or MAPKKK gene. Real-time PCR analyses were carried out according to the description by Wu et al. [46]. Two biological and at least three technical replicates for each sample were obtained in the real-time PCR machine (BIO-RAD CFX96, USA). To normalize the total amount of cDNA in each reaction, the tomato *SlUbi3* (accession number X58253) gene was co-amplified as an endogenous control to calibrate relative expression. The C_t_ method of relative gene quantification recommended by Applied Biosystems (PE Applied Biosystems, USA) was used to calculate the expression levels of different treatments. Student’s t-test was used to determine the statistical significance of the differential expression patterns between treatments. A heatmap was generated by matrix2png using the relative expression data of each SlMAPKK and SlMAPKKK gene [47].

## Results and Discussion

### Identification and sequence analysis of MAPKK and MAPKKK genes in tomato

The published tomato genome database enables the genome-wide analysis of the MAPKK and MAPKKK gene families in tomato [48]. To find all the members of these two families, BLASTP searches against a local database built using protein sequences were performed using these sequences, which contained 40 MAPKKs from four species, including *Arabidopsis*, rice, soybean, and *Populus trichocarpa*, and 155 MAPKKKs from *Arabidopsis*, rice, and maize. Only the members with above 50% identity were collected. Redundant sequences were removed manually. Thus, we found eight candidates for SlMAPKKs and 103 candidates for SlMAPKKKs. The candidate sequences were further evaluated by identifying the putative functional domains of through NCBI BLASTP (http://blast.ncbi.nlm.nih.gov/). The sequences without the relevant domains or conserved motifs were removed. After multiple cycles of these analyses, we identified five SlMAPKKs and 89 SlMAPKKKs from the currently available tomato. We completed EST hits and a full-length cDNA search to verify their existence (Tables 1 and 2). The existence of all MAPKKs was supported by EST hits except *SlMAPKK5,* and two out of five MAPKKs were found in full-length cDNA sequences. The existence of MAPKKK family genes was also supported by EST hits, whereas only 11 out of 89 SlMAPKKKs were found in full-length cDNA sequences. Given that no standard nomenclature is followed for MAPKKKs in plant species, we named them sequentially based on their distribution on chromosomes [5], [20].

**Table 1:** Characteristics of MAPK kinase (MAPKKs) from *Solanum lycopersicum*.

Gene	Deduced polypeptide			Total number ofmapped ESTs	Full-lengthcDNADDBJ Acc.Number	SGN ID	Predictedsubcellularlocation	Chromosomenumber	Location	Strand direction
	Length	Molecularweight (kDa)	PI							
SlMAPKK1	357	39.7	5.58	29	AK247428	Solyc12g009020.1.1	Cytoplasmic	12	2321616–2325502	–
SlMAPKK2	359	39.8	8.87	21	NA	Solyc03g123800.1.1	Nuclear	3	64579230–64580309	–
SlMAPKK3	354	39.6	5.89	7	AK322922	Solyc03g119490.2.1	Nuclear	3	62138086–62141226	+
SlMAPKK4	335	37.5	8.7	40	NA	Solyc03g097920.1.1	Mitochondrial	3	53756873–53757880	+
SlMAPKK5	515	57.5	5.49	0	NA	Solyc03g019850.2.1	Cytoplasmic	3	6757647–6762359	+

**Table 2:** Characteristics of MAPK kinase kinase (MAPKKKs) from *S. lycopersicum*.

Gene	Deduced polypeptide			Total no ofmapped ESTs	Full-lengthcDNA DDBJAcc.Number	SGN ID	PredictedSubcellularlocalization	Chromosomenumber	Location	Stranddirection
	Length	Molecularweight (kDa)	PI							
SlMAPKKK1	1152	131.0	6.18	14	AK320148	Solyc01g005030.2.1	Plasma Membrane	1	47011–54276	+
SlMAPKKK2	430	44.4	8.72	3	AK326321	Solyc01g010950.2.1	Cytoplasmic	1	6505612–6517771	–
SlMAPKKK3	760	85.1	6.52	2	NA	Solyc01g059860.2.1	Nuclear	1	61725129–61733391	–
SlMAPKKK4	688	75.9	7.22	1	NA	Solyc01g079750.2.1	Cytoplasmic	1	71407076–71414432	–
SlMAPKKK5	767	86.0	6.42	1	NA	Solyc01g096170.2.1	Nuclear	1	79028423–79033644	+
SlMAPKKK6	748	85.0	5.53	8	NA	Solyc01g097840.2.1	Nuclear	1	80304661–80308285	+
SlMAPKKK7	982	107.0	6.04	4	NA	Solyc01g097980.2.1	Nuclear	1	80387053–80397552	**+**
SlMAPKKK8	1618	180.0	8.60	0	NA	Solyc01g098980.2.1	Nuclear	1	81089216–81103000	–
SlMAPKKK9	359	39.6	5.34	7	NA	Solyc01g103240.2.1	Cytoplasmic	1	83654054–83655666	+
SlMAPKKK10	665	73.5	4.96	15	NA	Solyc01g104530.2.1	Nuclear	1	84745224–84751406	–
SlMAPKKK11	563	63.5	5.89	8	AK320250	Solyc01g111880.2.1	Cytoplasmic	1	89803298–89811106	–
SlMAPKKK12	1221	134.4	5.20	7	NA	Solyc02g031860.2.1	Nuclear	2	17793182–17800827	–
SlMAPKKK13	318	35.9	8.56	0	NA	Solyc02g064930.1.1	Mitochondrial	2	30606492–30607448	+
SlMAPKKK14	359	40.0	4.98	2	NA	Solyc02g064980.1.1	Cytoplasmic	2	30683256–30684335	–
SlMAPKKK15	630	70.8	8.73	5	NA	Solyc02g065110.2.1	Nuclear	2	30847497–30852710	–
SlMAPKKK16	461	52.4	5.72	4	NA	Solyc02g071740.2.1	Cytoplasmic	2	35686760–35693727	–
SlMAPKKK17	741	82.3	6.75	3	NA	Solyc02g076780.2.1	Nuclear	2	36457518–36480448	+
SlMAPKKK18	504	57.2	9.20	15	AK320304	Solyc02g078140.2.1	Nuclear	2	37494938–37497613	–
SlMAPKKK19	210	23.8	6.07	0	NA	Solyc02g087590.1.1	Cytoplasmic	2	44552788–44553420	–
SlMAPKKK20	638	70.5	9.12	10	NA	Solyc02g090430.2.1	Nuclear	2	46568708–46573422	+
SlMAPKKK21	360	40.1	5.02	3	NA	Solyc02g090970.1.1	Cytoplasmic	2	47002477–47003559	+
SlMAPKKK22	355	39.1	5.24	3	NA	Solyc02g090980.1.1	Cytoplasmic	2	47010791–47011858	+
SlMAPKKK23	356	39.6	5.33	1	NA	Solyc02g090990.1.1	Cytoplasmic	2	47018848–47019918	+
SlMAPKKK24	353	39.9	6.90	10	NA	Solyc02g093410.2.1	Cytoplasmic	2	48864525–48868381	+
SlMAPKKK25	480	54.5	9.38	4	NA	Solyc03g006400.2.1	Mitochondrial	3	996867–999265	–
SlMAPKKK26	890	95.1	9.38	1	NA	Solyc03g025360.2.1	Nuclear	3	7174598–7181522	+
SlMAPKKK27	664	74.0	4.86	6	NA	Solyc03g112140.2.1	Nuclear	3	56690058–56697440	+
SlMAPKKK28	351	39.8	8.41	0	NA	Solyc03g114310.2.1	Cytoplasmic	3	58374824–58379605	+
SlMAPKKK29	405	44.7	4.59	2	NA	Solyc03g117640.1.1	Chloroplast	3	60782770–60783987	–
SlMAPKKK30	1031	112.4	5.34	5	NA	Solyc03g119140.2.1	Nuclear	3	61887750–61899889	=
SlMAPKKK31	311	35.4	8.89	0	NA	Solyc03g121780.1.1	Nuclear	3	63894220–63896278	+
SlMAPKKK32	377	42.9	9.19	0	AK319354	Solyc04g014690.2.1	Nuclear	4	4951177–4954530	+
SlMAPKKK33	334	38.0	9.35	0	NA	Solyc04g064590.1.1	Nuclear	4	54872524–54874175	–
SlMAPKKK34	958	107.4	6.46	6	NA	Solyc04g076480.2.1	Nuclear	4	58934431–58942365	–
SlMAPKKK35	715	78.7	9.23	15	AK247731	Solyc04g079400.2.1	Nuclear	4	61503685–61508549	–
SlMAPKKK36	362	41.5	6.02	2	NA	Solyc05g041420.2.1	Cytoplasmic	5	50821713–50825381	+
SlMAPKKK37	913	98.1	9.33	3	NA	Solyc06g036080.2.1	Nuclear	6	22156308–22166784	–
SlMAPKKK38	426	47.4	5.05	0	NA	Solyc06g068510.1.1	Chloroplast	6	38853114–38854394	–
SlMAPKKK39	989	107.9	5.73	10	NA	Solyc06g068980.2.1	Nuclear	6	39180022–39189671	–
SlMAPKKK40	394	44.1	7.95	2	NA	Solyc06g071410.2.1	Nuclear	6	40320383–40324461	–
SlMAPKKK41	626	70.5	5.18	8	NA	Solyc06g071800.2.1	Nuclear	6	40604034–40613849	–
SlMAPKKK42	636	72.6	5.76	7	NA	Solyc06g082470.2.1	Nuclear	6	44554015–44559461	–
SlMAPKKK43	1083	120.4	5.28	9	NA	Solyc07g006760.2.1	Nuclear	7	1599263–1605156	–
SlMAPKKK44	1415	152.9	5.33	4	NA	Solyc07g007140.2.1	Nuclear	7	1889884–1899241	–
SlMAPKKK45	854	94.9	6.36	2	NA	Solyc07g008400.1.1	Plasma Membrane	7	3197916–3200480	–
SlMAPKKK46	412	46.2	7.68	29	AK31989	Solyc07g042680.2.1	Cytoplasmic	7	53543511–53549179	+
SlMAPKKK47	412	46.1	8.09	7	AK322903	Solyc07g042890.2.1	Nuclear	7	53735335–53740970	+
SlMAPKKK48	485	55.1	9.02	0	NA	Solyc07g047910.1.1	Mitochondrial	7	56433677–56437115	+
SlMAPKKK49	290	33.2	5.95	0	NA	Solyc07g047990.1.1	Cytoplasmic	7	56521372–56522845	+
SlMAPKKK50	326	36.4	5.85	0	NA	Solyc07g051860.1.1	Chloroplast	7	57721350–57722330	–
SlMAPKKK51	329	37.0	6.03	0	NA	Solyc07g051870.1.1	Mitochondrial	7	57725633–57726622	–
SlMAPKKK52	326	36.6	5.94	0	NA	Solyc07g051880.1.1	Chloroplast	7	57731960–57732940	–
SlMAPKKK53	329	36.7	5.78	0	NA	Solyc07g051890.1.1	Chloroplast	7	57754547–57755536	+
SlMAPKKK54	969	36.0	6.47	0	NA	Solyc07g051920.1.1	Chloroplast	7	57792333–57793301	+
SlMAPKKK55	370	41.2	5.95	0	NA	Solyc07g051930.1.1	Nuclear	7	57797942–57799054	+
SlMAPKKK56	601	66.1	5.93	3	NA	Solyc07g053170.2.1	Nuclear	7	58941217–58949834	–
SlMAPKKK57	813	91.1	5.86	2	NA	Solyc07g055130.2.1	Nuclear	7	60575268–60584663	–
SlMAPKKK58	459	112.2	5.06	0	NA	Solyc07g055870.2.1	Cytoplasmic	7	61111919–61118746	+
SlMAPKKK59	590	55.0	4.60	1	NA	Solyc07g064820.1.1	Plasma Membrane	7	64029373–64030845	–
SlMAPKKK60	304	35.3	5.56	1	NA	Solyc07g065250.2.1	Nuclear	7	64310077–64312539	–
SlMAPKKK61	723	81.0	6.29	1	NA	Solyc08g007910.2.1	Nuclear	8	2412322–2421171	+
SlMAPKKK62	445	51.2	6.45	0	NA	Solyc08g062140.1.1	Cytoplasmic	8	47692672–47697995	–
SlMAPKKK63	320	36.2	6.34	0	NA	Solyc08g069090.1.1	Plasma Membrane	8	55375982–55376944	–
SlMAPKKK64	677	42.9	9.28	8	NA	Solyc08g076490.2.1	Plasma Membrane	8	57660690–57663299	–
SlMAPKKK65	756	84.2	7.57	0	NA	Solyc08g080460.1.1	Nuclear	8	60917452–60922463	+
SlMAPKKK66	840	90.2	9.27	3	NA	Solyc08g081210.2.1	Nuclear	8	61435787–61442377	–
SlMAPKKK67	586	65.9	5.15	8	NA	Solyc08g082980.2.1	Nuclear	8	62771972–62775303	+
SlMAPKKK68	837	92.0	5.80	4	NA	Solyc09g009090.2.1	Nuclear	9	2427384–2442527	+
SlMAPKKK69	310	34.7	6.73	1	NA	Solyc09g018060.2.1	Cytoplasmic	9	12708472–12715748	–
SlMAPKKK70	606	69.5	5.09	17	AK321568	Solyc09g018170.2.1	Nuclear	9	13402635–13407530	+
SlMAPKKK71	731	83.1	5.13	14	NA	Solyc09g076000.2.1	Nuclear	9	63183204–63186781	+
SlMAPKKK72	322	36.3	5.76	7	AK324898	Solyc10g009060.1.1	Cytoplasmic	10	3091684–3092652	+
SlMAPKKK73	656	74.5	5.12	0	NA	Solyc10g009350.2.1	Nuclear	10	3414014–3418293	–
SlMAPKKK74	439	49.6	6.17	0	NA	Solyc10g017490.1.1	Cytoplasmic	10	5362924–5369956	+
SlMAPKKK75	563	63.7	5.83	19	AK321643	Solyc10g055720.1.1	Nuclear	10	52722218–52731615	–
SlMAPKKK76	525	59.6	5.92	16	NA	Solyc10g079130.1.1	Cytoplasmic	10	60064941–60068542	–
SlMAPKKK77	829	91.9	6.03	20	NA	Solyc10g083610.1.1	Nuclear	10	62712543–62724546	–
SlMAPKKK78	793	88.5	5.66	5	NA	Solyc10g085570.1.1	Nuclear	10	64008641–64021548	+
SlMAPKKK79	964	106.4	6.26	3	NA	Solyc10g085670.1.1	Plasma Membrane	10	64098873–64104331	–
SlMAPKKK80	614	67.0	9.19	9	NA	Solyc11g006000.1.1	Nuclear	11	809687–815717	+
SlMAPKKK81	374	42.8	9.00	0	NA	Solyc11g012050.1.1	Nuclear	11	4992438–4996469	+
SlMAPKKK82	1401	154.1	6.06	23	NA	Solyc11g033270.1.1	Nuclear	11	22947724–22983117	+
SlMAPKKK83	301	34.2	6.21	0	NA	Solyc12g005360.1.1	Plasma Membrane	12	218973–219878	+
SlMAPKKK84	400	44.8	7.16	5	NA	Solyc12g009340.1.1	Chloroplast	12	2619577–2623405	+
SlMAPKKK85	391	44.9	6.12	0	NA	Solyc12g013980.1.1	Cytoplasmic	12	4817640–4824070	+
SlMAPKKK86	362	40.8	8.26	11	NA	Solyc12g062280.1.1	Cytoplasmic	12	49627152–49631145	+
SlMAPKKK87	680	74.9	8.96	0	NA	Solyc12g088940.1.1	Nuclear	12	62566327–62571104	–
SlMAPKKK88	569	64.3	6.34	3	NA	Solyc12g094410.1.1	Cytoplasmic	12	62972424–62976753	+
SlMAPKKK89	466	52.5	5.91	5	NA	Solyc12g099250.1.1	Nuclear	12	64789614–64795487	–

The polypeptide lengths of the MAPKK genes ranged from 335 aa to 515 aa, and their predicted molecular weights ranged from 37.5 kD to 57.5 kD. The predicted pI has a range of 5.49 to 8.7. However, the polypeptide lengths of the MAPKKK genes ranged from 290 aa to 1618 aa, and their predicted molecular weights ranged from 23.8 kD to 180 kD. The theoretical pI has a range from 4.59 to 9.38 (Tables 1 and 2).

The MAPKK genes were predicted to be localized in the cytoplasm, nucleus, and mitochondria. Similarly, most of the MAPKKK genes were predicted to be localized in the cytoplasm, nucleus, and mitochondria, and others were predicted to be localized in the plasma membrane and chloroplast (Tables 1 and 2).

### Phylogenetic relationship, conserved domain, and gene structure analysis

**Figure 1:**
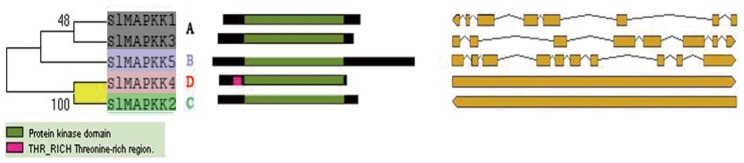
Phylogenetic analysis (left), domain organization (middle) and exon-intron structures (right) of tomato MAPKKs. The unrooted phylogenetic tree was generated using MEGA4.1 program by the neighbor-joining method. Bootstrap supports from 1000 replicates are indicated at each branch. The gene names of each subfamily are indicated with the same color. The domain organizations are analyzed by scanning of the protein sequences for the presence of known motifs and domains using PlantsP. The exon-intron organization of corresponding SlMAPKK genes is represented by yellow boxes and lines, respectively.

**Figure 2:**
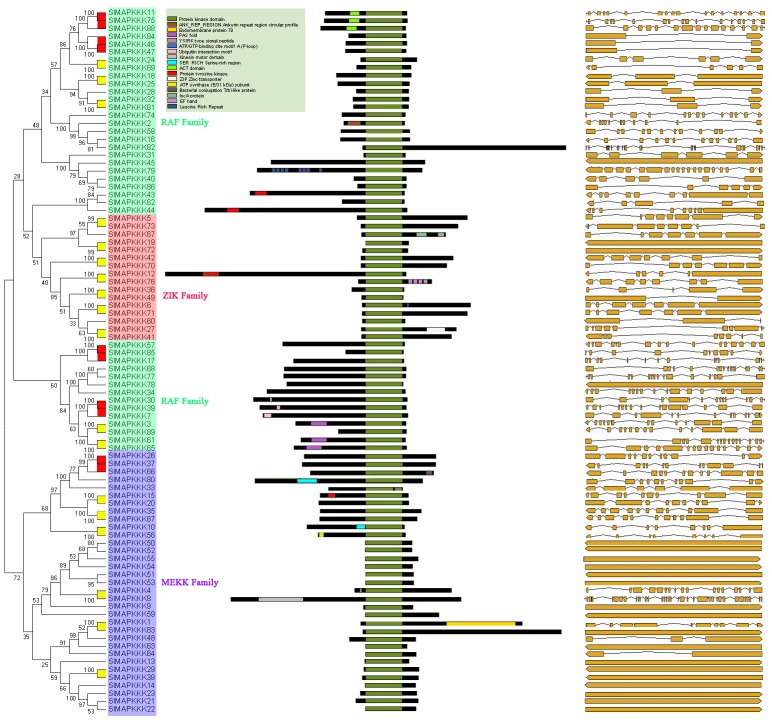
Phylogenetic analysis (left), domain organization (middle) and exon-intron structures (right) of 89 SlMAPKKKs in tomato. For other details, see Figure 1.

To further characterize the MAPKKs and MAPKKKs from tomato, the kinase domains of tomato were aligned using ClustalW and analyzed using MEGA4. Unrooted phylogenetic trees were generated from the alignment of the full-length protein sequences of all five SlMAPKKs and 89 SlMAPKKKs by the NJ and ME methods, and showed similar topologies with only minor modifications at deep nodes. Similar to those in *Arabidopsis* and rice [3], [4], five MAPKK genes in tomato formed four groups (groups A–D) (Fig. 1). Consistent with previous reports on *Arabidopsis*, rice, and maize [4], [20], [21], 89 SlMAPKKKs were divided into three categories, including 33 MEKK members, 16 ZIK members, and 40 RAF members (Fig. 2).

Conserved domain analysis showed a kinase domain in all the MAPKKs and MAPKKKs. In the SlMAPKKK family, most of the Raf family proteins contained a C-terminal kinase domain and long N-terminal regulatory domain except for *SlMAPK82*. By contrast, the majority of the ZIK members had an N-terminal kinase domain except for *SlMAPK12.* Protein tyrosine regions were distributed across different subfamily members. A ubiquitin-interaction motif and ACT domain functioning in the regulation of a wide range of metabolic enzyme activities were found only in the RAF subfamily (Fig. 2), which is consistent with the previous findings in rice and *Arabidopsis* [7], [20].

Based on the predicted sequences, tomato MAPKK and MAPKKK gene structures were mapped. *SlMAPKK1* and *SlMAPKK3* belonging to group A contained eight exons and seven introns, but MAPKK genes from groups C and D usually contained no intron (Fig. 1). The gene structures of MAPKKKs were highly divergent, even in the same subfamily. The intron-exon patterns of these genes showed no obvious similarity among the members even in the same group (Fig. 2). However, when comparing the intron-exon organization with phylogenetic analysis of these genes, we found that there was a relatively good correlation between intron conservation and phylogenetic relationships. Those genes clustering together on the phylogenetic trees often had similar intron-exon patterns (Figs.2). For example, *SlMAPKKK46*, *SlMAPKKK47*, and *SlMAPKKK84* clustered with very high bootstrap (100%) on the phylogenetic tree. Meanwhile, all of them contained only one intron (Figs. 1 and 2).

### Sequence alignments of conserved motifs

**Figure 3:**
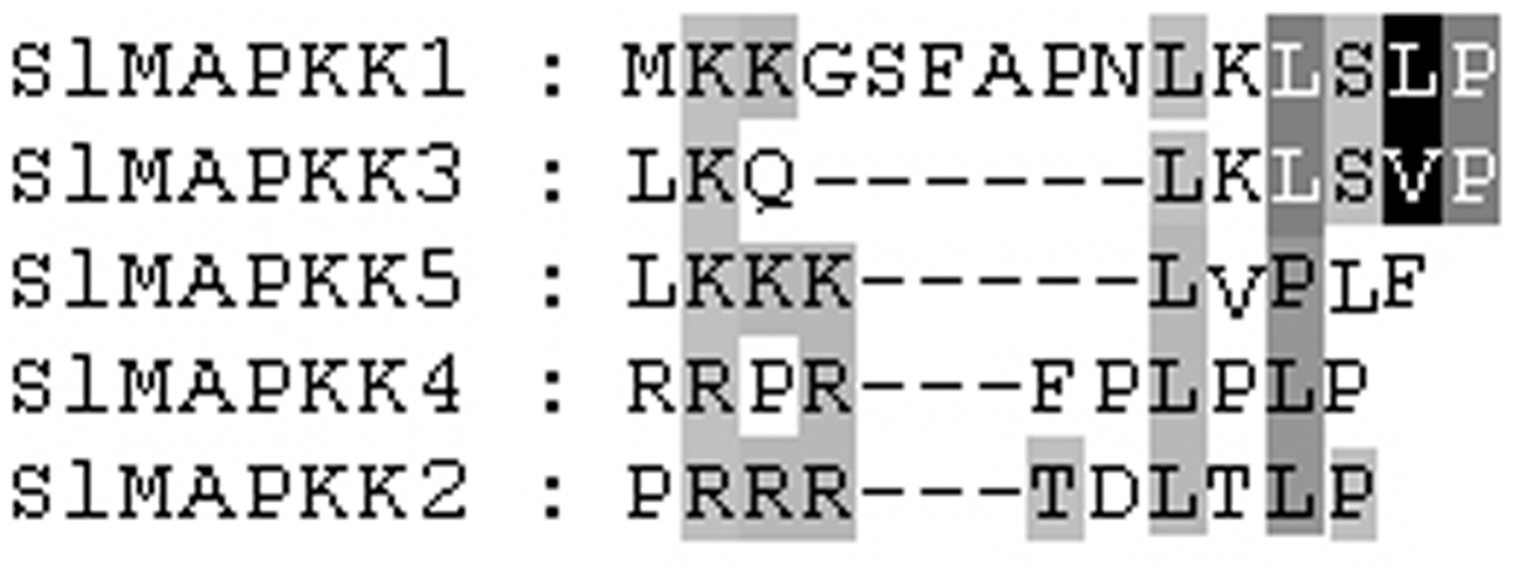
Alignment of SlMAPKK proteins in tomato. The highlighted part shows the conserved signature motif obtained with the ClustalX program.

All the identified MAPKK genes from *Arabidopsis*, rice, and poplar contain 11 catalytic subdomains [4], [49]. MAPKKs were also featured by a putative K/R-K/R-K/R-X (1-6)-L-X-L/V/I domain as a docking region. This conserved motif could also be found in most tomato MAPKK proteins (Fig. 3).

**Figure 4:**
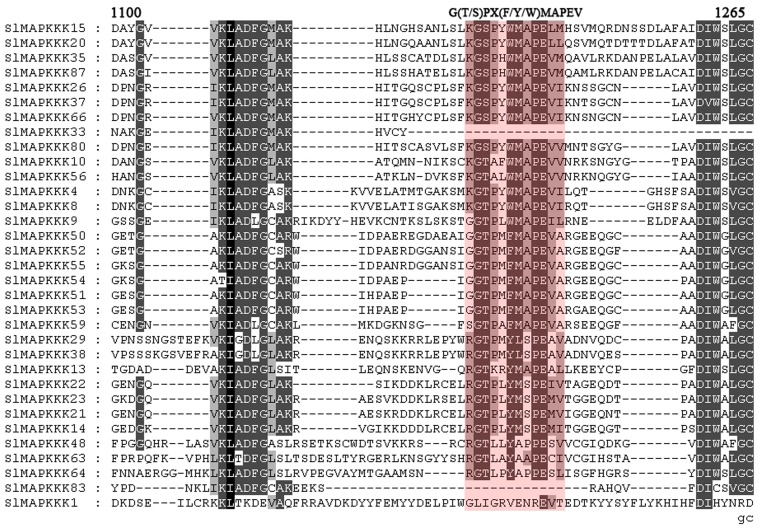
Alignment of MEKK-like SlMAPKKK proteins obtained with the ClustalX program. The highlighted part shows the conserved signature motif.

**Figure 5:**
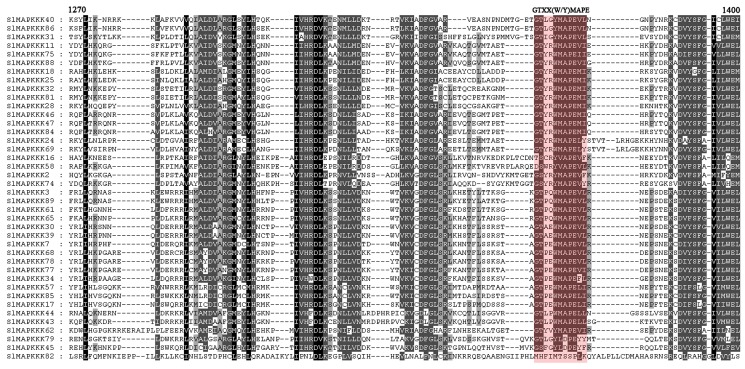
Alignment of Raf-like SlMAPKKK proteins obtained with the ClustalX program. The highlighted part shows the conserved signature motif.

**Figure 6:**
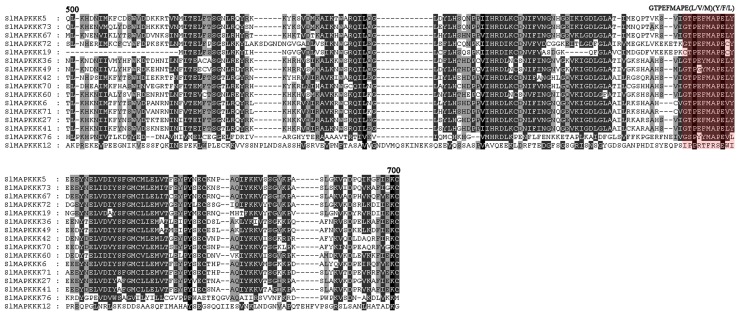
Alignment of ZIK-like SlMAPKKK proteins obtained with the ClustalX program. The highlighted part shows the conserved signature motif.

The plant MAPKKK gene family was formed by three subfamilies, each of which contains signature sequences or motifs different from those in the other two subfamilies [7]. In this study, a conserved motif G (T/S) PX (F/Y/W) MAPEV [20] was found in all 33 putative MEKK genes except *SlMAPKKK1*, *SlMAPKKK33*, and *SlMAPKKK83*, further confirming that they belonged to the MEKK subfamily (Fig. 4). The RAF family is the largest subfamily in tomato and other reported species with a conserved signature GTXX (W/Y) MAPE in its kinase domain across the members [20]. In tomato, this signature was also found in all the members of the RAF family except SlMAPK83, strongly supporting their identity as members of the RAF subfamily (Fig. 5). The characteristic feature of the ZIK family consists of a conserved signature GTPEFMAPE (L/V/M) (Y/F/L) across the members [20]. Sixteen MAPKKKs out of 89 members had ZIK specific signatures (Fig. 6).

### Chromosomal mapping and gene duplication

**Figure 7:**
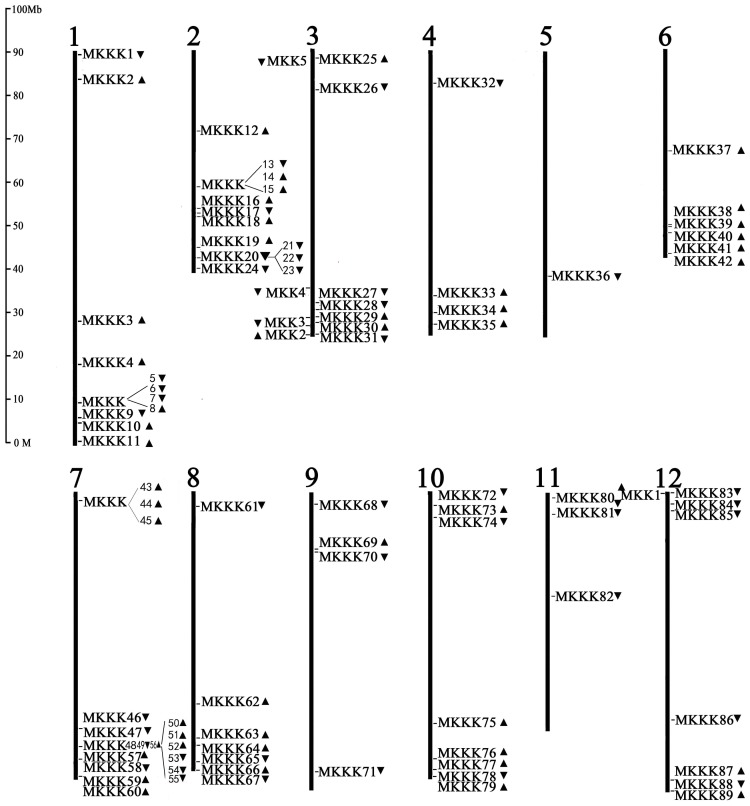
Chromosomal distribution of SlMAPKKs and SlMAPKKKs genes in tomato genome. The names of each tandem duplicated gene cluster of the two families were indicated with black rectangles. The triangles indicate the upward or downward direction of transcription.

The chromosomal locations and transcription directions of tomato *MAPKK* and *MAPKKK* genes were determined and demonstrated using BLASTN analysis on tomato WGS chromosomes. Interestingly, five SlMAPKKs were distributed on chromosomes 3 and 12 (Fig. 7). Four of them were located on chromosome 3, and the other one was located on chromosome 12. Although the SlMAPKKK family genes were distributed over all the 12 chromosomes (Fig. 7), the number in each chromosome differed, ranging from one (chromosome 5) to 18 (chromosome 7).

Gene duplication events have an important function in the amplification of gene family members in tomato genome. Gene families can arise through the tandem amplification or segmental duplication of chromosomal regions [50]. In this study, no tandem duplicated gene pairs and segmental duplicate families were found in the SlMAPKK gene. In the SlMAPKKK gene family, we found two clusters (Fig. 7, red box) and 23 segmental duplications. Synteny analysis further confirmed the segmental duplications among the SlMAPKKK genes. Most of the pairs of segmental duplicates were distributed on different chromosomes. Three pairs were also distributed on the same chromosome. Even though some gene pairs shared high similarity in sequence, such as *SlMAPKK2/SlMAPKK4*, *SlMAPKKK1/SlMAPKKK83*, *SlMAPKKK10/SlMAPKKK56*, *SlMAPKKK57/SlMAPKKK85*, and *SlMAPKKK7/SlMAPKKK30/SlMAPKKK39*, we found no clear evidence of segmental duplication among them (Fig. S1). Thus, the expansion of the MAPKKK gene families in tomato might be a consequence of whole genome or chromosomal segment duplications. The tandem duplications may have slight affection.

### Cis-elements in promoter regions of SlMAPKK and SlMAPKKK genes

Genes responsive to multiple stimuli are closely correlated with cis-regulatory elements in their promoter regions [43]. To further understand transcriptional regulation and the potential function of SlMAPKKs and SlMAPKKKs, cis-elements in their promoter sequences were predicted. Many cis-elements involved in plant growth and resistance were found in the 2 kb upstream region of tomato *SlMAPKK* and *SlMAPKKK* genes using the PLACE database (http://www.dna.affrc.go.jp/PLACE/) (Tables S2 and S3). One salt-stress (S000453), one heat-stress (S00030), one cold-stress (S000407), one wound-stress (S000457), three drought-stresses (S000176, S000407, and S000409), and disease resistance (S000024)-related cis-elements were all found in the promoter regions of both *SlMAPKKs* and *SlMAPKKKs*. Moreover, auxin (S000273), GA (S000259), ABA (S000292), and ET (S000037) signaling transduction-related cis-elements were found in most of the detected sequences (Tables S2 and S3). These results suggest that most SlMAPKKs and SlMAPKKKs may participate in tomato development and in response to stressful environments.

### Evolutionary patterns and divergence of MAPKK and MAPKKK genes in plants

To further investigate the evolutionary relationships of MAPKK and MAPKKK proteins, we compared these two gene families between two monocotyledonous (maize and rice) and two dicotyledonous plants (*Arabidopsis* and tomato). Unrooted phylogenetic trees were constructed based on 37 MAPKK and 318 MAPKKK sequences (Figs. S2 and S3). The numbers of MAPKKs and MAPKKKs in different species are indicated in Table 3. The AtMAPKKs and AtMAPKKKs were downloaded from TAIR. The OsMAPKKs and OsMAPKKKs were downloaded from KOME. The ZmMAPKKKs were downloaded from NCBI. Given that the ZmMAPKKs have not been reported systematically, we identified and analyzed MAPKK family genes in maize using the same method for identifying tomato SlMAPKKs (Table S4, Figs. S4 and S5).

**Table 3:** The numbers of SlMAPK, SlMAPKK, and SlMAPKKK in *Arabidopsis*, rice, tomato, and maize.

Species	MAPK	MAPKK	MAPKKK			Total number of MAPKKKs
			MEKK	ZIK	RAF	
*Arabidopsis*	20	10	21	11	48	80
tomato	16	5	33	16	40	89
rice	15	8	22	10	43	75
maize	20	14	22	6	46	74

Similar to previous studies [3], [4], 37 MAPKKs were divided into four groups (groups A–D) (Fig. S2). However, no maize MAPKK genes belonged to groups B and C (Figs. S2 and S4), thereby implying that groups B and C MAPKK proteins might be lost in the maize genome after species differentiation. Although group D of MAPKKs contained genes from all four species, more than half (12 out of 20) came from maize (Figs. S2 and S4). This result indicates that the members of group D may have more important functions in maize than in other species, and gene expansion in this group could lead to a large maize MAPKK gene family.

All the 318 MAPKKKs from four different species formed three subfamilies, namely, MEKK, RAF, and ZIK (Fig. S3). Most of the groups or subfamilies contained members from all four species (Table 3, Fig. S3), implying that the genes within these classes were derived from a common ancestor. However, the number of SlMAPKKKs in the MEKK and ZIK subfamilies was larger than that in the same subfamilies in the other three species (Table 3), which indicates that gene expansion of tomato MAPKKKs mainly occurred in these two subfamilies.

### Expression profile of SlMAPKK and SlMAPKKK genes in different tissues or organs

**Figure 8:**
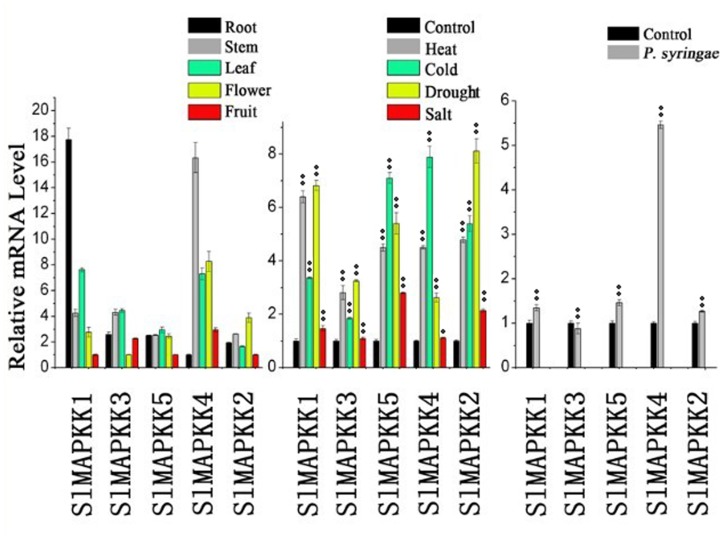
Expression profiles of SlMAPKK family genes in tomato using qRT-PCR analysis. A: transcript levels of all 5 SlMAPKK in different tomato organs including root, stem, leaf, flower buds, and fruit. B: transcript level change of all 5 SlMAPKK genes in tomato seedlings exposed to heat (H), Cold (C), drought (D), salt (S) stresses when compared to control treatment. C: transcript levels of all five SlMAPKK genes in tomato seedlings exposed to *Pseudomonas syringes*. Data represent the means and standard errors of three independent biological samples. Relative expression levels were normalized relative to a reference gene *SlUbi3* (accession number X58253). Asterisks indicate significant differences as determined by Student’s t-test (*P, 0.05; **P, 0.01).

Expression analysis of SlMAPKKs revealed that most of these genes were constitutively expressed because their expression could be detected in most selected organs (Fig. 8). Relatively higher expression levels for *SlMAPKK1* and *SlMAPKK4* than those for other SlMAPKKs were found in tomato organs. The expression pattern of these two genes is different from that of their orthologs in *Arabidopsis* (MPSS database) and soybean [5]. The expression levels changed markedly among different organs/tissues. For example, *SlMAPKK1* and *SlMAPKK4* had the highest expression values in the root and stem, respectively, whereas *SlMAPKK2* had a relatively high expression level in the flower.

**Figure 9:**
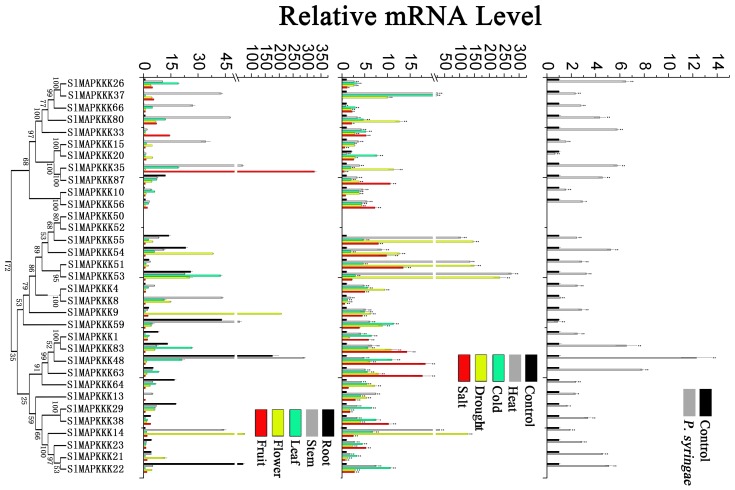
Expression patterns of MEKK subfamily genes in different organs and under abiotic and biotic stress treatment in tomato by qRT-PCR analysis. For other details, see Figure 8.

**Figure 10:**
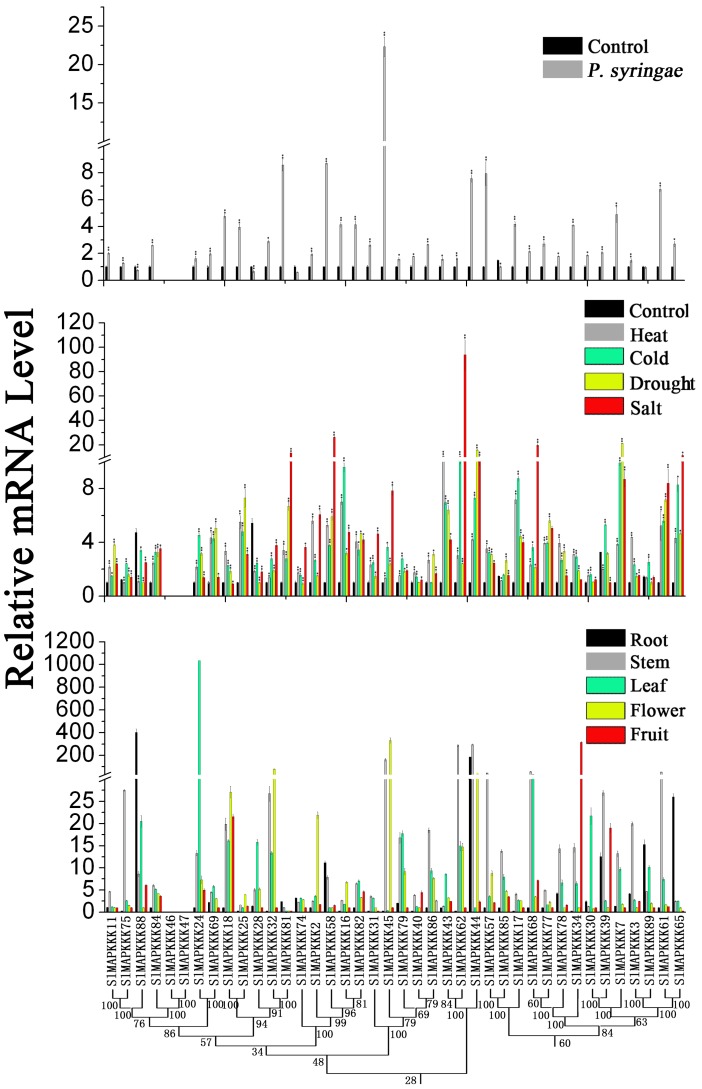
Expression patterns of RAF subfamily genes in different organs and under abiotic and biotic stress treatment in tomato by qRT-PCR analysis. For other details, see Figure 8.

**Figure 11:**
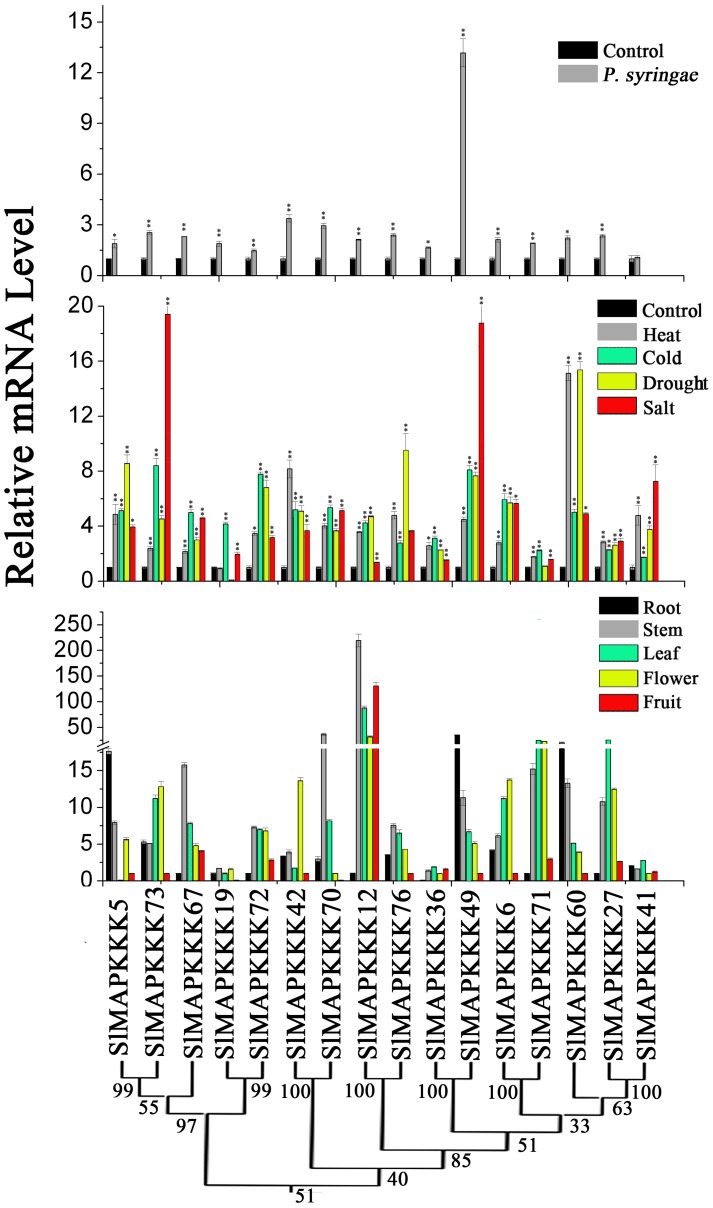
Expression patterns of ZIK subfamily genes in different organs and under abiotic and biotic stress treatment in tomato by qRT-PCR analysis. For other details, see Figure 8 (Left corresponding to upper part; middle corresponding to middle part; Right corresponding to lower part).

No specific primers could be found to distinguish corresponding genes from each other because of the high similarity in nucleotide sequence between *SlMAPKKK46* and *SlMAPKKK4* and between *SlMAPKKK50 a*nd *SlMAPKKK52*. Thus, the expression patterns of all the tomato MAPKKK genes, except above four SlMAPKKK genes were analyzed. The expression of most SlMAPKKK genes was detected in all the selected organs (Figs. 9, 10, and 11). However, some genes were highly expressed in one or several specific organs. Twelve SlMAPKKKs from three subfamilies had higher expression levels in the root than that in other organs (Figs. 9, 10, and 11). Meanwhile, 13 SlMAPKKKs belonging to three subfamilies showed markedly higher expression levels in tomato stem than that in other organs (Figs. 10 and 11). Only three SlMAPKKKs (*SlMAPKKK33*, *SlMAPKKK34*, and *SlMAPKKK35*) were expressed with high abundance in fruits, while they were clustered at the end of chromosome 4 (Fig. 10).

### Expression patterns under various stress conditions

MAPKK genes in plants are involved in response to various biotic and abiotic stresses. In *Arabidopsis*, *MAPKK2* has an important function in the cold and salt signaling transduction pathway [13], [14]. In maize, *ZmMAPKK4* is essential for salt and cold tolerance because the overexpression of *ZmMKK4* in *Arabidopsis* leads to insensitivity to salt and cold treatment [51]. Both *AtMAPKK1* and *AtMAPKK2* are associated with plant innate immunity [13]–[15]. In addition, *AtMAPKK3* has a function in pathogen resistance [17], [18]. In tomato, *SlMAPKK2* acts with *SlMPK2,* thereby directly contributing to resistance to *Xanthomonas campestris* pv. vesicatoria [52]. In this study, the relative mRNA level of five SlMAPKKs changed significantly under heat, cold, drought, and salt stresses (Fig. 8). All five SlMAPKK genes were upregulated by heat, cold, and drought treatment, whereas *SlMAPKK2* and *SlMAPKK5* were significantly upregulated by salt stress (Fig. 8). By contrast, the expression level of *SlMAPKK4* was also dramatically upregulated (more than fourfold) after *P. syringae* treatment (Fig. 8), which indicates that *SlMAPKK4* may also have an important function in the defense response to tomato pathogens.

The expression pattern of tomato MAPKKK genes under abiotic and biotic treatment was also analyzed in detail. Most of the SlMAPKKK genes were significantly upregulated by all four abiotic treatments (Figs. 9, 10, and 11), namely, heat, cold, drought, and salt. The relative mRNA levels of *SlMAPKKK51*, *SlMAPKKK53*, and *SlMAPKKK55* were upregulated by more than 100-fold after heat or drought treatment (Fig. 9). Meanwhile, 13 MAPKKK genes showed a more than 10-fold change in expression levels under salt treatment (Figs. 9, 10, and 11). These data indicate that most *S*lMAPKKK genes were involved in the regulation of various abiotic stress signaling transduction pathways. After *P. syringae* treatment, some SlMAPKKK genes were also remarkably upregulated. Especially for *SlMAPKKK45*, *SlMAPKKK48*, and *SlMAPKKK49*, the relative mRNA levels were increased by more than 10-fold after treatment (Figs. 9, 10, and 11), indicating that these SlMAPKKK genes may have special functions in plant pathogen resistance.

The expression patterns of MAPKKK duplicated gene pairs were also investigated. Only three pairs (*SlMAPKKK12* and *SlMAPKKK76*, *SlMAPKKK6* and *SlMAPKKK71*, and *SlMAPKKK4* and *SlMAPKKK8*) and one paralogous pair, including *SlMAPKKK1*, *SlMAPKKK83*, and *SlMAPKKK84*, shared similar expression patterns in nearly all stress conditions, whereas other paralogs were different. Although the duplicated SlMAPKKK genes had high similarity in amino acid sequences, they may have evolved a different expression pattern and function. Similar observations on the plant MAPKKK family have also been reported in maize [21] and soybean [5].

### Expression profiles under IAA and SA treatment

**Figure 12:**
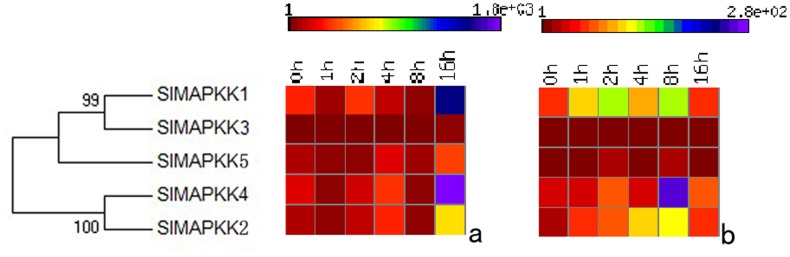
Heat map shows the real-time quantitative RT-PCR (qRT-PCR) analysis results of *SlMAPKK* genes with exogenous IAA (left) and SA (right) treatments.

MAPK cascades interact with or participate in the signal transduction of many plant hormones, such as auxin, ethylene, abscisic acid, SA, and jasmonic acid (JA) [32], [53]–[55]. In this study, the expression patterns of tomato MAPKK and MAPKKK genes after exogenous IAA and SA treatment were analyzed in detail. All the SlMAPKKs except *SlMAPKK3* and *SlMAPKK5* were upregulated in response to IAA and SA treatment (Fig. 12). In *Arabidopsis*, *MKK7* negatively regulates polar auxin transport and subsequently affects plant architecture [56]. In tobacco, the overexpression of *SIPK* enhances ozone-induced ethylene formation and blocks ozone-induced SA accumulation [57]. JA can activate the MAPK cascade *MKK3-MAPK6* and negatively regulate *ATMYC2/JIN1* expression, thereby controlling *Arabidopsis* root growth [18]. All these results provide clear evidence of the involvement of the MAPKK pathway in response to hormone treatments.

**Figure 13:**
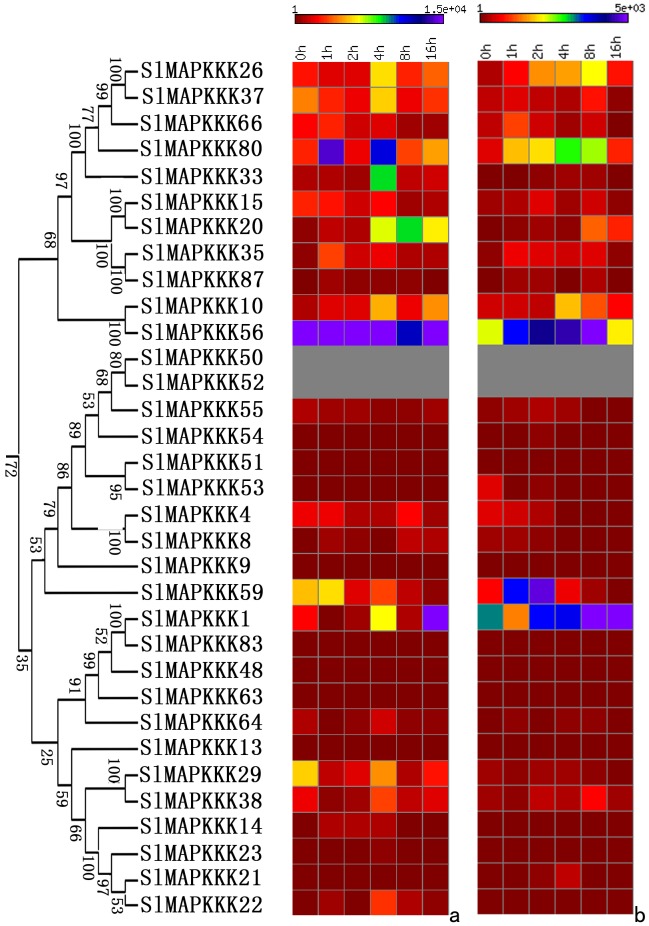
Expression profiles of MEKK subfamily genes with exogenous IAA (left) and SA (right) treatments.

**Figure 14:**
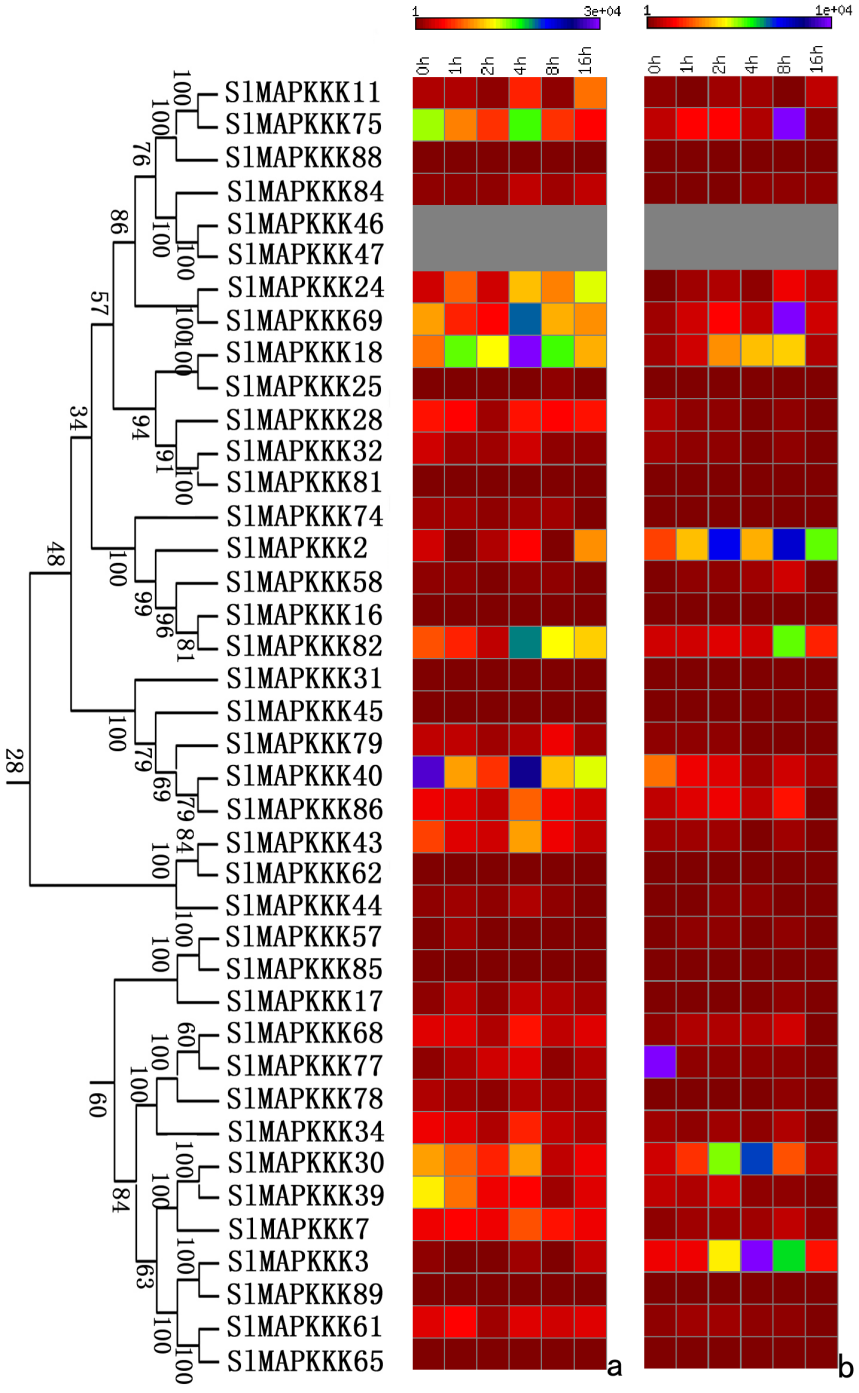
Expression profiles of RAF subfamily genes with exogenous IAA (left) and SA (right) treatments.

**Figure 15:**
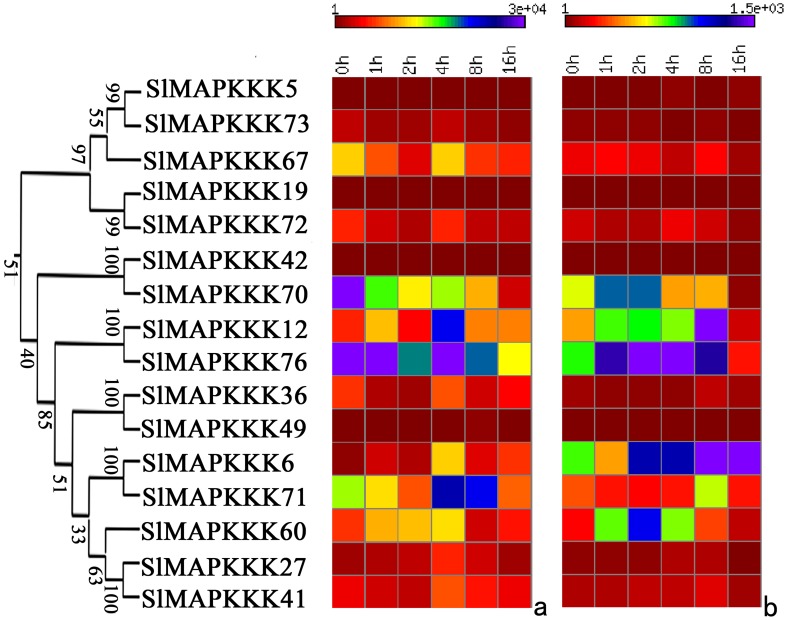
Expression profiles of ZIK subfamily genes with exogenous IAA (left) and SA (right) treatments.

The mRNA levels of most SlMAPKKKs varied considerably at different time periods after exogenous IAA and SA treatment (Figs. 13, 14, and 15). Almost half of the MEKK subfamily members were markedly upregulated by IAA and SA treatment, whereas others showed nearly no change with a relatively low expression level (Fig. 13). The RAF subfamily genes shared similar expression patterns with the MEKK subfamily (Fig. 14). However, all the ZIK subfamily members, except *SlMAPKKK5*, *SlMAPK42*, and *SlMAPK49*, had a remarkable response after IAA and SA treatment (Fig. 15). These data imply that most SlMAPKKK genes may be involved in plant hormone signaling during plant development and defense response. In rice, a MAPK gene, *BWMK1*, responds to other plant hormones, such as JA, SA, and benzothiadiazole [58]. Using the *Arabidopsis* leaf protoplast transient expression system, Kovtun et al. proved that an oxidative stress MAPK cascade can negatively regulate early auxin response [59]. However, evidence of the involvement of MAPKKK in hormonal responses is limited. The patterns of interaction between the MAPK cascades and hormone signaling pathway need further investigation [60].

### Conclusion

MAPK cascade family genes should be systematically analyzed to understand their functions in plant development and stress response. In this study, we present the genome-wide identification and analysis of the MAPKK and MAPKKK gene families in tomato. Five SlMAPKKs and 89 SlMAPKKKs were identified from the available tomato genome. Based on structural characteristics and a comparison of phylogenetic relationships among tomato, *Arabidopsis*, maize, and rice, all these MAPKK and MAPKKK genes were divided into four and three groups, respectively. Our results suggest that chromosomal segment duplications may be the main factors for the expansion of the MAPKKK gene family in tomato. Although nearly all the MAPKK and MAPKKK family genes were expressed in all the detected organs, some genes were highly expressed in one or several specific organs. The expression of most SlMAPKKs and SlMAPKKKs could be induced by both abiotic and biotic stress treatment. Most of the SlMAPKK and SlMAPKKK genes may interact with plant hormones, such as auxin and SA, during plant development and defense pathways. Our study could help improve the understanding of the complexity of the MAPKK cascade and guide future studies for functional analyses. The functions of organ-specific and stress-related genes in MAPK cascades and interaction with other signaling pathways in tomato are being characterized in our laboratory using overexpression and knockdown methods.

## Supporting Information

Figure S1 **Synteny analysis of SlMAPKKK genes in ±100kb region.**
(DOCX)

Figure S2 **The phylogenetic tree of MAPKK genes from** ***Arabidopsis*** **, tomato, rice, and maize.**
(TIF)

Figure S3 **The phylogenetic tree of MAPKKK genes from** ***Arabidopsis*** **, tomato, rice, and maize.**
(TIF)

Figure S4 **Phylogenetic analysis (Left), domain organization (middle) and exon-intron structures (right) of maize. ZmMAPKK genes.**
(TIF)

Figure S5 **The feature domain of ZmMAPKK proteins obtained with the ClustalX program.**
(TIF)

Table S1 **Primer sequences of SlMAPKK and SlMAPKKK genes for qRT-PCR expression analysis.**
(DOC)

Table S2 **The cis-elements in promoter sequences of MAPKK genes in tomato.**
(XLSX)

Table S3 **The cis-elements in promoter sequences of MAPKKK genes in tomato.**
(XLS)

Table S4 **The characteristics of MAPKK family genes in maize.**
(XLSX)
