# Supplementary material for: Genome-Wide Identification of MAPKK and MAPKKK Gene Families in Tomato and Transcriptional Profiling Analysis during Development and Stress Response
Source: PLoS One. 2014 Jul 18;9(7):e103032. doi: 10.1371/journal.pone.0103032 (PMC4103895; doi:10.1371/journal.pone.0103032)
Supplement: Figure S1 — Synteny analysis of SlMAPKKK genes in ±100kb region. (DOCX) [file pone.0103032.s001.docx]

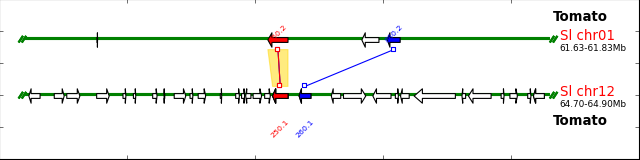

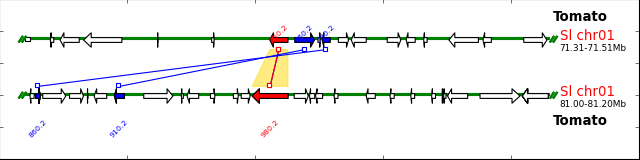

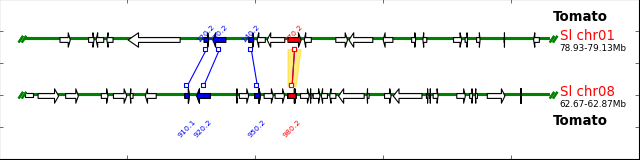


***SlMAPKKK5***

***SlMAPKKK67***

***SlMAPKKK4***

***SlMAPKKK8***

***SlMAPKKK3***

***SlMAPKKK89***


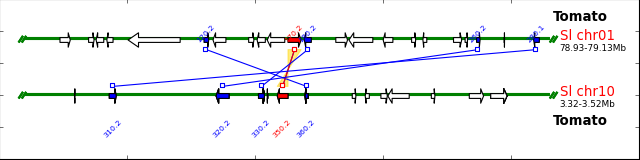

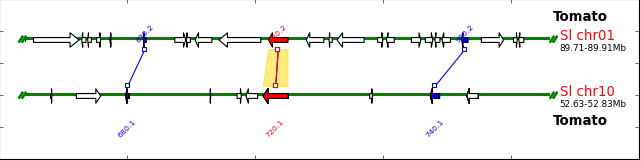

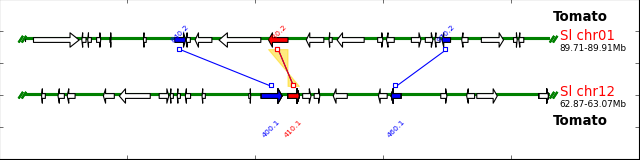


***SlMAPKKK11***

***SlMAPKKK88***

***SlMAPKKK5***

***SlMAPKKK73***

***SlMAPKKK11***

***SlMAPKKK75***


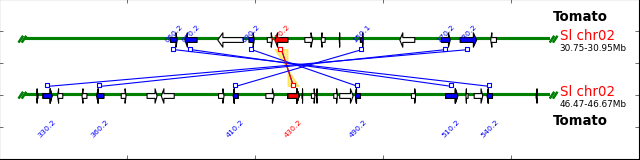


***SlMAPKKK15***

***SlMAPKKK20***


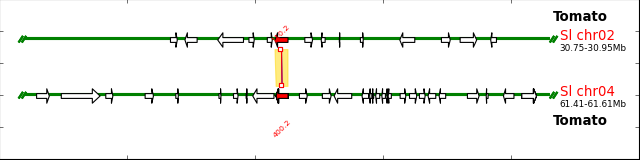


***SlMAPKKK15***

***SlMAPKKK35***


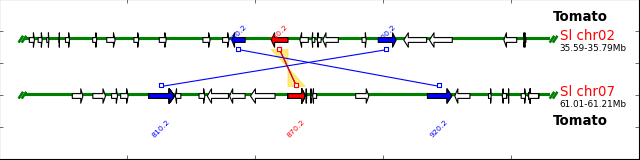


***SlMAPKKK16***

***SlMAPKKK58***


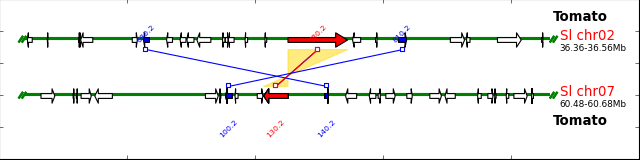


***SlMAPKKK17***

***SlMAPKKK57***


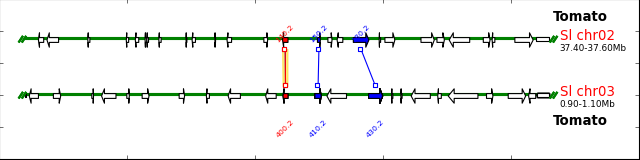


***SlMAPKKK18***

***SlMAPKKK25***


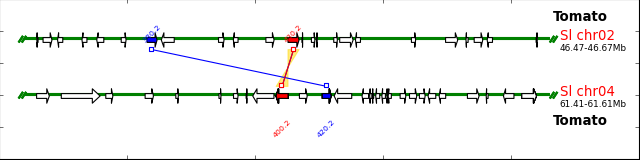

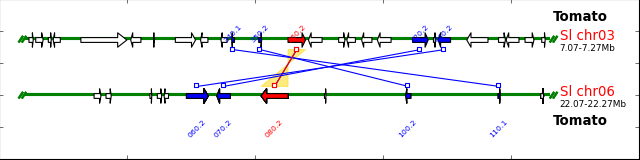

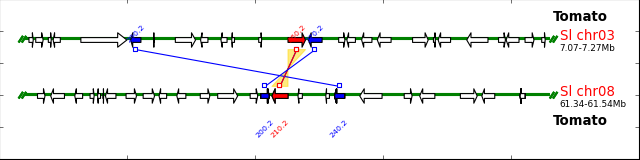


***SlMAPKKK26***

***SlMAPKKK66***

***SlMAPKKK26***

***SlMAPKKK37***

***SlMAPKKK20***

***SlMAPKKK35***


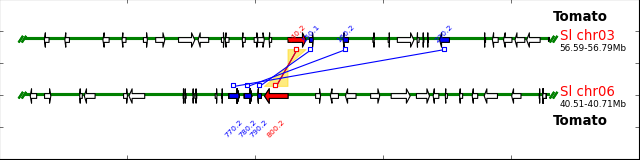


***SlMAPKKK29***

***SlMAPKKK38***

***SlMAPKKK27***

***SlMAPKKK41***


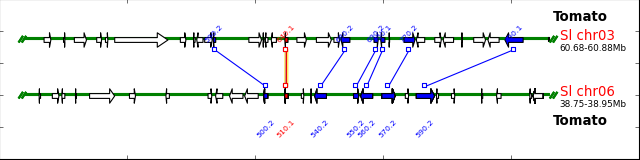

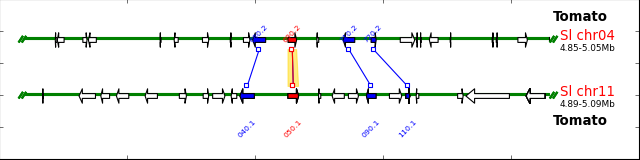

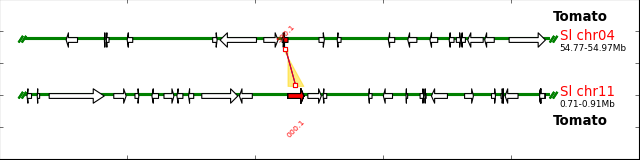

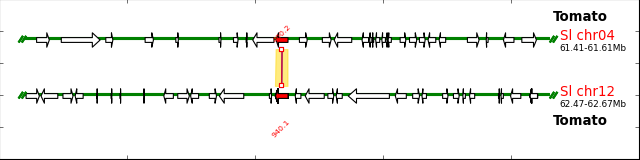

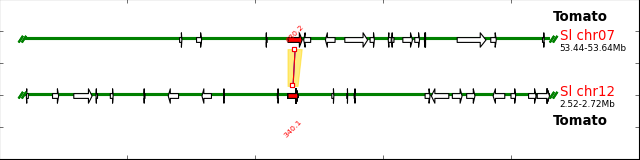

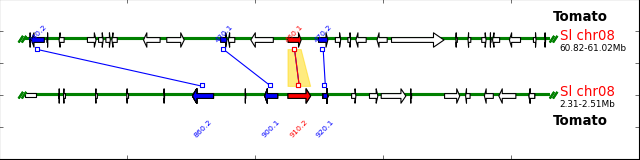


***SlMAPKKK61***

***SlMAPKKK65***

***SlMAPKKK46***

***SlMAPKKK84***

***SlMAPKKK35***

***SlMAPKKK87***

***SlMAPKKK33***

***SlMAPKKK80***

***SlMAPKKK32***

***SlMAPKKK81***


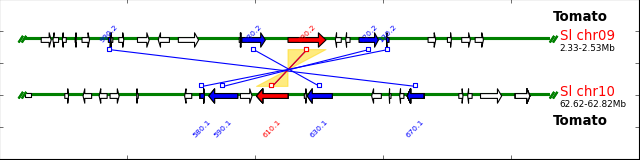

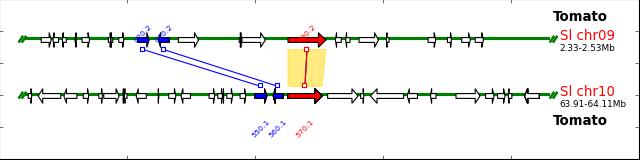


***SlMAPKKK68***

***SlMAPKKK78***

***SlMAPKKK68***

***SlMAPKKK77***

Fig S1.Synteny analysis of *SlMAPKKK* genes in ±100kb region.Synteny analysis revealed evidence of the segmental duplications among *SlMAPKKK* genes.
