# Supplementary figures and images for: Genome-Wide Identification of MAPKK and MAPKKK Gene Families in Tomato and Transcriptional Profiling Analysis during Development and Stress Response
Source: PLoS One. 2014 Jul 18;9(7):e103032. doi: 10.1371/journal.pone.0103032 (PMC4103895; doi:10.1371/journal.pone.0103032)

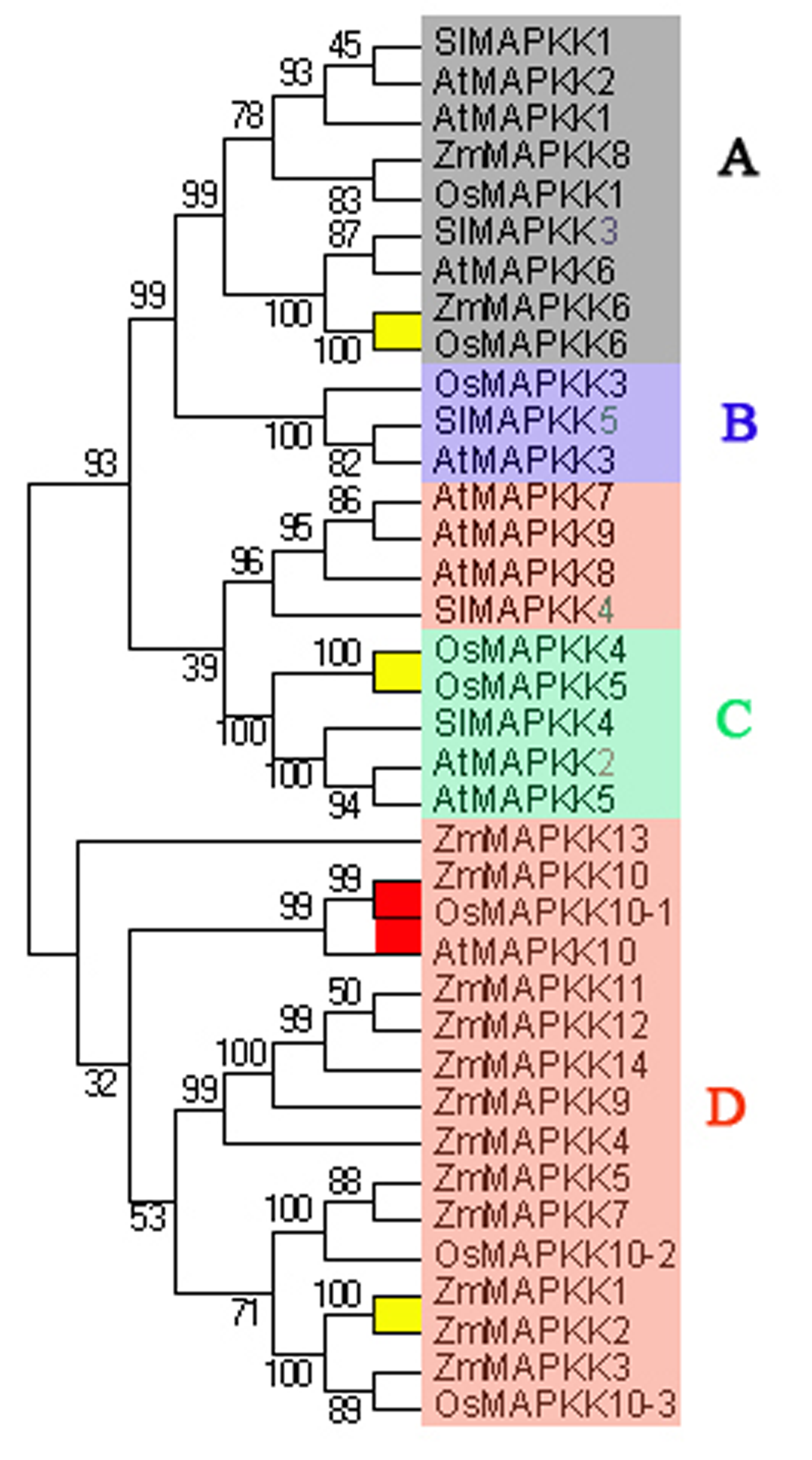

Supplement: Figure S2 — The phylogenetic tree of MAPKK genes from Arabidopsis , tomato, rice, and maize. (TIF) [file pone.0103032.s002.tif]

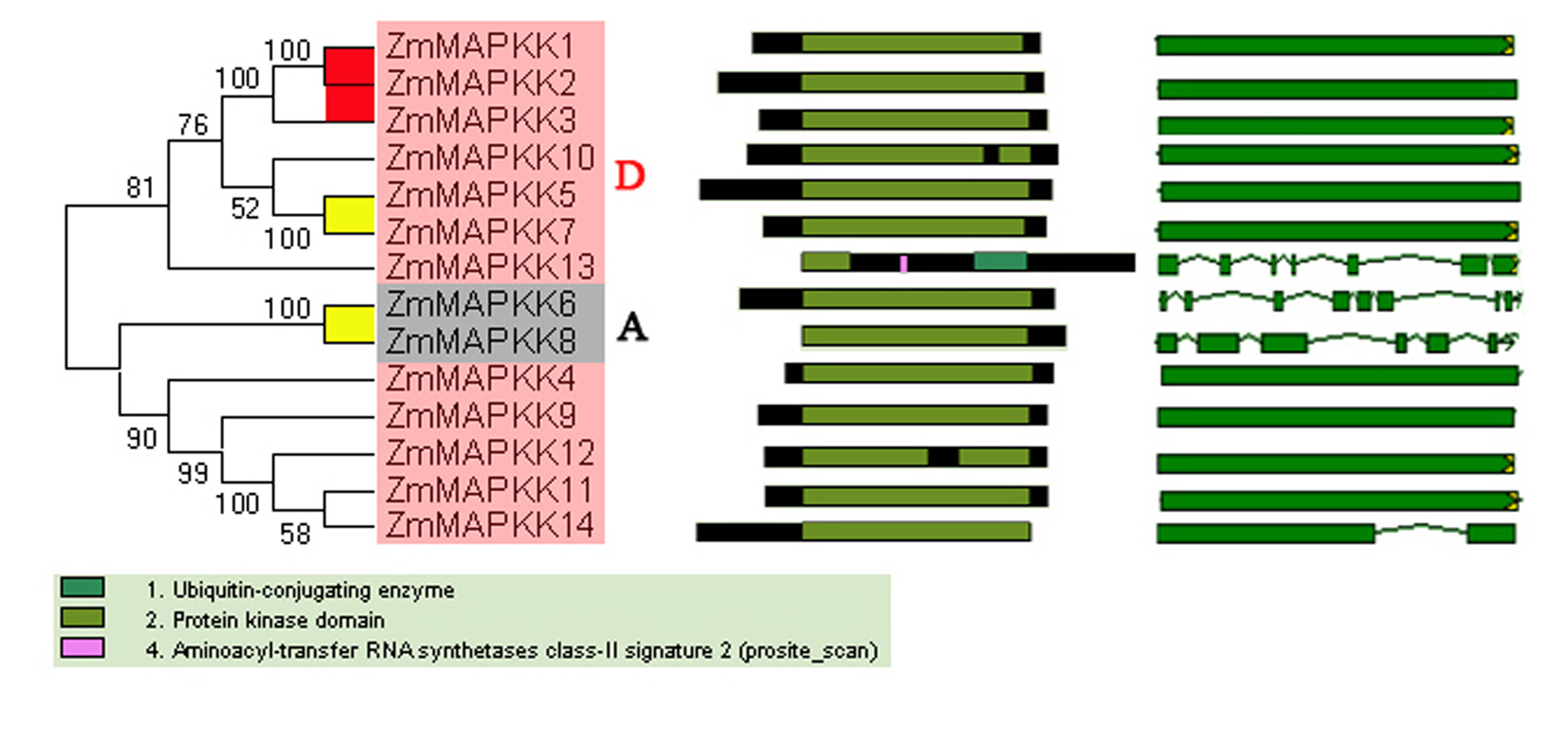

Supplement: Figure S4 — Phylogenetic analysis (Left), domain organization (middle) and exon-intron structures (right) of maize. ZmMAPKK genes. (TIF) [file pone.0103032.s004.tif]

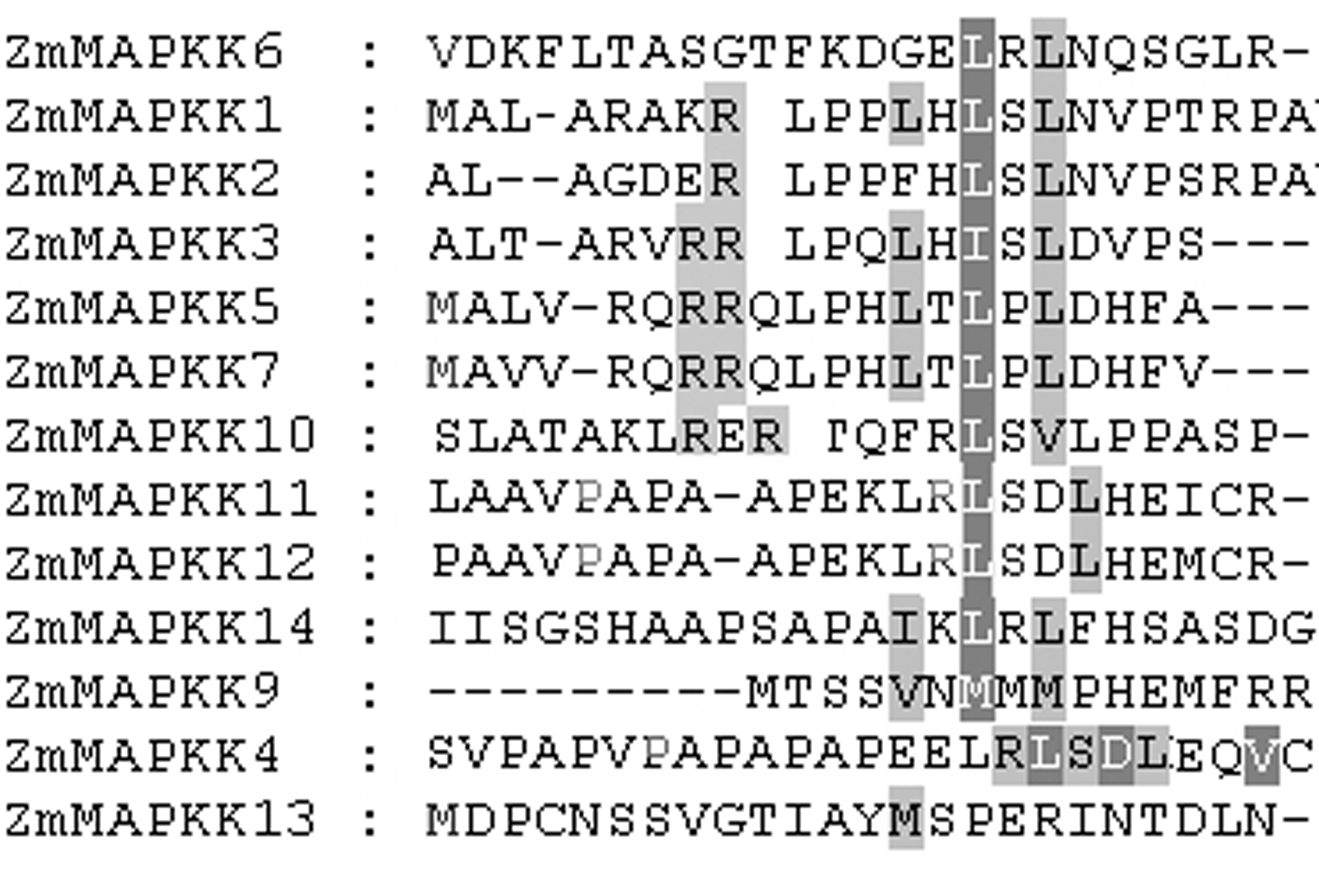

Supplement: Figure S5 — The feature domain of ZmMAPKK proteins obtained with the ClustalX program. (TIF) [file pone.0103032.s005.tif]
