# Supplementary material for: Genome-Wide Identification of MAPKK and MAPKKK Gene Families in Tomato and Transcriptional Profiling Analysis during Development and Stress Response
Source: PLoS One. 2014 Jul 18;9(7):e103032. doi: 10.1371/journal.pone.0103032 (PMC4103895; doi:10.1371/journal.pone.0103032)
Supplement: Table S1 — Primer sequences of SlMAPKK and SlMAPKKK genes for qRT-PCR expression analysis. (DOC) [file pone.0103032.s006.doc]

| Gene name | Forword(5’-3’) | Reverse(5’-3’) |
| --- | --- | --- |
| SlMAPKK1 | AAGCACCAGAAGGACAGAC | CCTGCGGAAGTGAAGTAAG |
| SlMAPKK2 | TTTGTATGTCTCAGCCTCC | GGTGCTATTCTGGGTGATG |
| SlMAPKK3 | TTTGTATGTCTCAGCCTCC | GGTGCTATTCTGGGTGATG |
| SlMAPKK4 | CTCTGCCCTCAGAAACTAAG | CAACCCATTTATGACGAAC |
| SlMAPKK5 | GCCAGCAAATCTACTTGTC | CTCATTCCGTATTCGTTCC |
| SlMAPKKK1 | GTCCTGGCATTATCAAGAAG | GCTGTAAGTAGGCTAAACGC |
| SlMAPKKK2 | GTGCTGGGCTAAAGATGTG | TCTGAAGGTAACCCTAGTTTG |
| SlMAPKKK3 | GACGTTAAGCATACAGAAGC | CAGTGGATCAAGTCCAAGTG |
| SlMAPKKK4 | GCACAAAGGATGGATGTTAG | TCCCTAGTTTCCAAAGTGAC |
| SlMAPKKK5 | GGTAGAGGACGAGCATTTC | CCACATGAGGACCAGTTATC |
| SlMAPKKK6 | TGAGCAGGAAATGAGATGG | GTGGAAGTCCCAAGTGTTG |
| SlMAPKKK7 | TTGGACTTCACCGTTAGC | CAACCTCAAGCCATCACC |
| SlMAPKKK8 | GCTTACTGCGGGTGACTG | CTCGCCTTCAACTGCTATC |
| SlMAPKKK9 | GTTAGCAGATTTGGGATGTG | GTGCCACCAGTAGACTTTG |
| SlMAPKKK10 | GGTTTCAGACCACCAAGG | ATCTTCATCATCGGCAGG |
| SlMAPKKK11 | GGCAATAAAGGATAACGAG | CTCCGAAGCACCTCATTG |
| SlMAPKKK12 | CGGCCTTAATCGTTTATC | ATGATCTGTTGTGAGCCTTC |
| SlMAPKKK13 | TGTATCAATGGCTTCTACTGC | GAGCGATTCAACAAACTAGAC |
| SlMAPKKK14 | GGATGAACTCAATGGGTG | GCTAACGAACCTCCAGAAG |
| SlMAPKKK15 | CTTTCAATGGTGGAGCAAC | ATCATCCTGGACAATGGAG |
| SlMAPKKK16 | TGTTGGGTAAGCAATCGTC | CCCTCATTAGCAGAATACATC |
| SlMAPKKK17 | CCGAAGATAAAGCAAACTC | GTGTCCCACAGAATCTGAG |
| SlMAPKKK18 | CCTACGGCATTTCACATC | AACTCACCCTCCCAACAC |
| SlMAPKKK19 | CAGAAGAGTTGCTGAAAGATC | TTGCATGACTAGTAAGAATATTG |
| SlMAPKKK20 | GTGGAAAGATCGCAAGTG | GAGGAAGTGGTAGTGGATG |
| SlMAPKKK21 | GATTCCGAGAAACCAGAGTAC | GTTCATCAAAGTAGCTGAGC |
| SlMAPKKK22 | GTCCAATTACATCTTTGCC | GCACCACTTAACAGTGCC |
| SlMAPKKK23 | TGGTGGTCAAGTCATCTGC | CATCGAATAATGTAAGGGC |
| SlMAPKKK24 | GCCTGTCATGGTCATAGTAAC | CAAGGGCAAATCTAATAGC |
| SlMAPKKK25 | CTAGACCTGGAACGACTTCTC | AAAGCCCTCTGCTTCTTTG |
| SlMAPKKK26 | CCAAATATTTCTCCACCTGC | GATATTCCTTGCCTGTCCAA |
| SlMAPKKK27 | GGTGATGGTGCCTTTGAG | TTTGCCCTGACGAATGAG |
| SlMAPKKK28 | TACAAGCACAGGGAAGTGG | TTAACCGAAGCAACAAAGC |
| SlMAPKKK29 | GATGATTCCCTTGGTTACTG | TCAGAACCACCATCAATGG |
| SlMAPKKK30 | CCTCCTTTGGCTTATCATG | GCTGCCCACTAACTTCTCC |
| SlMAPKKK31 | TATTGGTTACATGGCTCCG | CACAAGCAAATTCCAAAGC |
| SlMAPKKK32 | ACCGTTATCAGTTCCTTCC | CATTGACCATGACTCCAAAC |
| SlMAPKKK33 | TCATCCCCTACCTCATCTC | TGACCAGTCATTGCATTTG |
| SlMAPKKK34 | CAGACTTACAAGAAGGAGGTTG | TTGTCGGATGTTTCTCACG |
| SlMAPKKK35 | CGGTTAGAGTTGACAGGGAG | CGCCGATAAGTTGGAGTAG |
| SlMAPKKK36 | CTGCCATTGCTACAATCAC | TCAGTCGCTTCAGGATTTG |
| SlMAPKKK37 | AAGATGCCTTCATGGTGG | CGGAGACTTAGCTTCAGC |
| SlMAPKKK38 | GAAAGGATGAGCATTAGAAGC | TGATAGATTCCGTGAAGCTAC |
| SlMAPKKK39 | CAGTCTGAAGTAGCTCCACC | CAACTACCATCGAAGAGGC |
| SlMAPKKK40 | GAGCAAGAACTATGGACAAGG | GCCACTGTAGCAATAGAAATG |
| SlMAPKKK41 | CTGACCGATTCACCAACTC | CTTCGTGTCCGAGTAAGG |
| SlMAPKKK42 | GTTGTTGCACAAGTCATTG | CCTCTTCAAATCCTCGTAAG |
| SlMAPKKK43 | GTGTTCGTGGCACTTTACC | GTGAGCAACTCCCACATAAC |
| SlMAPKKK44 | GTTGCCTCACAGATTCAGC | AAGCATTGTACCCTTGGTC |
| SlMAPKKK45 | CAAGTAATGCCACAGATGC | CAGAAATCAATGAAGGGTC |
| SlMAPKKK48 | CACATCAGTGAAGAAGCGTAG | ATGCCCAAATATCAACACC |
| SlMAPKKK49 | AAGTGTTGATTCGATGGAG | GCACCATGACCTAGTAACTC |
| SlMAPKKK51 | CAGAAACGATGTATTCCAC | CTCCAATTACATTCTCCTG |
| SlMAPKKK53 | AGAGGAATCAGAATCTACGAC | GCTCTAATTCACTCGAATTC |
| SlMAPKKK54 | GGACTAGAGTTCCTACATTCAG | GCACCGGATTTACCTAAC |
| SlMAPKKK55 | GGGTAAGTAAATTGAGCCAG | CTCTGTTACAGCTTTCCAGG |
| SlMAPKKK56 | CTGTATCAGATTGGAAAGGG | CTTGCAGACAGTGGTTAATG |
| SlMAPKKK57 | GCAGTATCATTACCCTCGTC | TCTTATTCCAGGTTGAGACC |
| SlMAPKKK58 | CGCTGGGTAGACAATCATC | CCAAATCACCGTCATTAGTC |
| SlMAPKKK59 | TGAGATGGCTACAGGATTAG | TCATCAAGCATTTACCGAG |
| SlMAPKKK60 | GAGTGTGATAACGTGGTGAAG | TTGAAGGAGTTCAGAAGCAG |
| SlMAPKKK61 | CCTCTGTTTCAAATCTGGC | AAATCAGCCTCATCCCTAG |
| SlMAPKKK62 | TAACAGGAGAAACAGGCAC | GCTCATTGAGAATAATGGC |
| SlMAPKKK63 | GCCCGTAGATAGAAACAAG | AAGTCCTTTGCTTCCTCTG |
| SlMAPKKK64 | CAGAAATCTTGACAACCCTC | GTTGAGACATTACCGTTATCG |
| SlMAPKKK65 | GCTACCTGCTCCAGAAAG | GACTTCCGTGACTGTTGG |
| SlMAPKKK66 | GATCCCTACATCTCCTAGACAG | TTTCATGCCCAAATACCC |
| SlMAPKKK67 | TTCCGAGGTTCACTTGTTG | TGTTCACATCGTCCACCC |
| SlMAPKKK68 | GTATTACATGCCGTCGTTG | CATCCTCACTTCAGCACC |
| SlMAPKKK69 | CGGGAAGTTGTTATGATGTC | CAGGCAGTAGCTCTGAAAC |
| SlMAPKKK70 | GGGATGAATGAAATGCTTG | CTATCTTGGTTGCTCCTTTC |
| SlMAPKKK71 | GAGAATGAGATACGACGAGAG | TTGCTGGACCTTTGAGTTG |
| SlMAPKKK72 | TCTCCCTGTGGTAGGTTTG | CAATTTGCTCAACACGATC |
| SlMAPKKK73 | GGAATCCGTCTTCTGGTG | GTACGGGCAACCACTCAC |
| SlMAPKKK74 | TAGGAGGACCATCAAATAGG | TCTTGTGCCAGCATTCAC |
| SlMAPKKK75 | GTGGTAGTAGAGTGGTGGATTC | TCATCGTTATCAGATTCCTTG |
| SlMAPKKK76 | TACAGATTCTTATGGATGTTGG | CAGGTGCTCCTCATTTGC |
| SlMAPKKK77 | TCGGATCATTTCGGGTAC | CACAGCCCTCATTCTTATG |
| SlMAPKKK78 | CAGCAGCAACCACCATAC | TCAAGTCCCAATATAATCCC |
| SlMAPKKK79 | TCAGACAGCTCAAAGGGTC | CATCGCTCTTCTCGGTTAG |
| SlMAPKKK80 | GTTCTCCACCCAGTCCTTC | CCTGTTGAATCCAAGATACAC |
| SlMAPKKK81 | CCTTTTAGGCTAAGAAGGTC | TCCATATCACAAGAACTCCAC |
| SlMAPKKK82 | GAACATCTACAGCGAGCAG | TGCTCCTGTCTTCTCTTATTG |
| SlMAPKKK83 | GATGAATGCTCTGTTCTACG | GTGCTTTGGTCTTCTCCG |
| SlMAPKKK84 | CAATTAAGCAGGCCTTAC | TTGTCCGATTTCAGATCC |
| SlMAPKKK85 | GCACTCAAGCAGAACCGAC | TGAACTTATCTGCTCCATC |
| SlMAPKKK86 | TTATAGGAGCTGCAATGGG | GGAAGGTATTCAACCACTACAC |
| SlMAPKKK87 | ACAAGTCCGAATCTTAGCC | ATCTATCCGAAGAAGTGGC |
| SlMAPKKK88 | GCCTAAATGAAGATATGCG | CCAATAAACTGCACAACATTC |
| SlMAPKKK89 | GCTCAGTGAGCCACAATC | GAATAACGTACTGCCTCTGTAG |
